# Multigene Phylogeography of *Bactrocera caudata* (Insecta: Tephritidae): Distinct Genetic Lineages in Northern and Southern Hemispheres

**DOI:** 10.1371/journal.pone.0129455

**Published:** 2015-06-19

**Authors:** Hoi-Sen Yong, Phaik-Eem Lim, Ji Tan, Sze-Looi Song, I Wayan Suana, Praphathip Eamsobhana

**Affiliations:** 1 Institute of Biological Sciences, University of Malaya, 50603 Kuala Lumpur, Malaysia; 2 Chancellory High Impact Research, University of Malaya, 50603 Kuala Lumpur, Malaysia; 3 Institute of Ocean and Earth Sciences, University of Malaya, 50603 Kuala Lumpur, Malaysia; 4 Department of Agricultural and Food Science, Faculty of Science, Universiti Tunku Abdul Rahman, Jalan Universiti Bandar Barat, 31900, Kampar, 31900 Perak, Malaysia; 5 Faculty of Science and Mathematics, Mataram University, Mataram, Indonesia; 6 Department of Parasitology, Faculty of Medicine Siriraj Hospital, Mahidol University, Bangkok 10700, Thailand; Sichuan University, CHINA

## Abstract

*Bactrocera caudata* is a pest of pumpkin flower. Specimens of *B*. *caudata* from the northern hemisphere (mainland Asia) and southern hemisphere (Indonesia) were analysed using the partial DNA sequences of the nuclear 28S rRNA and internal transcribed spacer region 2 (ITS-2) genes, and the mitochondrial cytochrome *c* oxidase subunit I (COI), cytochrome *c* oxidase subunit II (COII) and 16S rRNA genes. The COI, COII, 16S rDNA and concatenated COI+COII+16S and COI+COII+16S+28S+ITS-2 nucleotide sequences revealed that *B*. *caudata* from the northern hemisphere (Peninsular Malaysia, East Malaysia, Thailand) was distinctly different from the southern hemisphere (Indonesia: Java, Bali and Lombok), without common haplotype between them. Phylogenetic analysis revealed two distinct clades (northern and southern hemispheres), indicating distinct genetic lineage. The uncorrected ‘p’ distance for the concatenated COI+COII+16S nucleotide sequences between the taxa from the northern and southern hemispheres (‘p’ = 4.46-4.94%) was several folds higher than the ‘p’ distance for the taxa in the northern hemisphere (‘p’ = 0.00-0.77%) and the southern hemisphere (‘p’ = 0.00%). This distinct difference was also reflected by concatenated COI+COII+16S+28S+ITS-2 nucleotide sequences with an uncorrected 'p' distance of 2.34-2.69% between the taxa of northern and southern hemispheres. In accordance with the type locality the Indonesian taxa belong to the nominal species. Thus the taxa from the northern hemisphere, if they were to constitute a cryptic species of the *B*. *caudata* species complex based on molecular data, need to be formally described as a new species. The Thailand and Malaysian *B*. *caudata* populations in the northern hemisphere showed distinct genetic structure and phylogeographic pattern.

## Introduction

*Bactrocera* (*Zeugodacus*) *caudata* (Fabricius 1805) is a pest of pumpkin infesting the host at the flowering stage [1]. It has a Paleartic and Oriental distribution, occurring in India, Sri Lanka, Myanmar, Thailand, Vietnam, China, Malaysia, Brunei and Indonesia (Sumatra, Java, Flores) [2].

*B*. *caudata* is easily recognized by the possession of three yellow vittae on the thorax, a transverse black band across the furrow of the face, two pairs of scutellar bristles and a slightly enlarged costal band at the apex of the wing [1,2]. It was first described from Java, Indonesia [1].

In a study of *B*. *caudata* from Peninsular Malaysia based on 14 gene-enzyme systems with 17 loci, the proportion of polymorphic loci was P = 0.41 and the mean heterozygosity was H = 0.11 [3]. Most of the molecular and phylogenetic studies involving *B*. *caudata* used only a single individual and from a single locality, e.g. Ranong, Thailand [4], Brunei [5], and Chongqing region, China [6]. Our recent study, using the partial DNA sequences of cytochrome *c* oxidase subunit I (COI) and 16S rRNA genes, revealed that *B*. *caudata* from Peninsular Malaysia was distinctly different from *B*. *caudata* of Bali and Lombok, without common haplotype between them [7]. Phylogenetic analysis revealed two distinct clades, indicating distinct genetic lineage. However, the study did not include the nominal taxon from the type locality Java, Indonesia.

The present study examined the partial DNA sequences of the nucler 28S rRNA and ITS-2 genes, and the mitochondrial COI, COII and 16S rRNA genes in several populations of *B*. *caudata* from mainland Asia (Malaysia and Thailand) and *B*. *caudata* from Indonesia (Java, Bali and Lombok) to determine their taxonomic and phylogenetic relationships.. We report here the genetic relationships of the nominal species from Java, Indonesia to the other taxa from various geographical regions. The Indonesian taxon is distinctly different from that of the northern hemisphere.

## Materials and Methods

### Ethics statement

*Bactrocera* fruit flies are pests of agricultural crops. They are not endangered or protected species. No permits are required to study these insects.

### Specimens, DNA Extraction, Polymerase Chain Reaction, and Sequencing

Male *Bactrocera* fruit flies were collected by application of cue-lure on the surface a green leaf. They were collected by means of specimen tubes, brought back to the laboratory and placed in deep freezer for about 10 min. The flies were then preserved in absolute ethanol and stored in deep freezer until use for DNA extarction. Flies were identified according to existing literature [1,7]. *Bactrocera tau* (Walker) was used as an outgroup. Details of the taxa studied and GenBank accession numbers of representative sequences of the haplotypes found in this study are listed in Table 1.

**Table 1:** Samples of *Bactrocera* fruit flies and gene markers used in this study.

Species	Voucher	Locality	Molecular Marker /Haplotype						
			COI	COII	16S	28S	ITS-2	CombinedCOI+COII+16S	CombinedCOI+COII+16S+28S+ITS-2
*B*. cf *caudata*	Bcau1	University Malaya,Kuala LumpurP.Malaysia	C1	D1;**KP694329**	R1	E1;**KP694325**	I1;**KP694336**	M1	N1
*B*. cf *caudata*	Bcau2	University Malaya,P. Malaysia	JN542417C1	D2	R1;JN542423	E1	I1	M2	N2
*B*. cf *caudata*	Bcau3	Clearwater,Perak,P.Malaysia	C1	D2;**KP694330**	R1	E1	I2;**KP694337**	M2	N3
*B*. cf *caudata*	Bcau4	Clearwater,Perak,P.Malaysia	C1	D2	R1	E1	I1	M2	N2
*B*. cf *caudata*	Bcau5	Clearwater,Perak,P.Malaysia	C1	D1	R1	E1	I1	M1	N1
*B*. cf *caudata*	Bcau7	Dungun,Terengganu,P..Malaysia	C1	D2	R1	E1	I1	M2	N2
*B*. cf *caudata*	Bacu8	Clearwater,Perak,PMalaysia	C1	D2	R1	E1	I1	M2	N2
*B*. cf *caudata*	Bcau9	Mentakab,Pahang,P.Malaysia	C1	D2	R1	E1	I1	M2	N2
*B*. cf *caudata*	Bcau10	Clearwater,Perak,P.Malaysia	C1	D2	R1	E1	I1	M2	N2
*B*. cf *caudata*	Bcau11	Carey Island,Selangor,P.Malaysia	C1	D2	R1	E1	I2	M2	N3
*B*. *caudata*	Bcau12	Sekotong,Lombok,Indonesia	C2;JN542418	D3;**KP694331**	R2;JN542424	E1	I1	M3	N4
*B*. *caudata*	Bcau13	Sekotong,Lombok,Indonesia	C2	D3	R2	E1	I1	M3	N4
*B*. cf *caudata*	Bcau14	Carey Island,SelangorP.Malaysia	C1;JN542416	D2	R3;JN542422	E1	I2	M4	N5
*B*. *caudata*	Bcau15	Tabanan, Bali,Indonesia	C2;JN542419	D3	R2;JN542425	E1	I1	M3	N4
*B*. cf *caudata*	Bcau16	Gombak,Selangor,P.Malaysia	C1	D1	R1	E2;**KP694326**	I2	M1	N6
*B*. *caudata*	Bcau17	Gili Meno, Lombok,Indonesia	C2	D3	R2	E1	I1	M3	N4
*B*. cf *caudata*	Bcau18	University Malaya,P. Malaysia	C1	D1	R1	E2	I1	M1	N8
*B*. cf *caudata*	Bcau19	Penang, P.Malaysia	C1	D1	R1	E2	I1	M1	N8
*B*. cf *caudata*	Bcau20	Penang, P. Malaysia	C1	D2	R1	E1	I2	M2	N3
*B*. cf *caudata*	Bcau21	Penang, P. Malaysia	C1	D1	R1	E1	I2	M1	N7
*B*. cf *caudata*	Bcau22	Pulau Tioman,Pahang,P. Malaysia	C1	D1	R1	E1	I2	M1	N7
*B*. cf *caudata*	Bcau23	University Malaya,P. Malaysia	C1	D4;**KP694332**	R1	E1	I1	M5	N9
*B*. cf *caudata*	Bcau24	Pulau Tioman,Pahang,P.Malaysia	C3;**KP694327**	D6;**KP694333**	R1	E1	I2	M6	N10
*B*. cf *caudata*	Bcau25	Korat, Thailand	C4;**KP694328**	D5;**KP694334**	R1	E2	I2	M7	N11
*B*. cf *caudata*	Bcau26	Gombak,Selangor,P.Malaysia	C1	D1	R1	E2	I1	M1	N8
*B*. cf *caudata*	Bcau27	Selakan, Sabah, E.Malaysia	C1	D1	R1	E1	I1	M1	N1
*B*. cf *caudata*	Bcau28	Semporna, Sabah,E.Malaysia	C1	D1	R1	E1	I1	M1	N1
*B*. *caudata*	Bcau29	Malang,Java,Indonesia	C2	D3	R2	E1	I1	M3	N4
*B*. *caudata*	Bcau30	Malang, JavaIndonesia	C2	D8;**KP694335**	R2	E1	I1	M8	N12
*B*. *caudata*	Bcau31	Malang, Java,Indonesia	C2	D3	R2	E1	I1	M3	N4
*B*. cf *caudata*	Bcau32	Jemaluang, Johor, P.Malaysia	C1	D1	R1	E1	I1	M1	N1
*B*. cf *caudata*		Ranong, Thailand	C1;AF423109	-	-	-	-	-	-
*B*. cf *caudata*		Lukut, Negeri Sembilan,Malaysia	C1;FJ903493	-	-	-	-	-	-
*B*. cf *caudata*		Chongqing, China	C1;GQ458048	-	-	-	-	-	-
*B*. cf *caudata*		Bandar Seri Begawan,Brunei	-	-	R1;AY037363	-	-	-	-
*B*. cf *caudata*		Bandar Seri Begawan, Brunei	-	D7;AY037406	-	-	-	-	-
Outgroup*B*. *tau*	Btau28	University Malaya,P. Malaysia	Not applicable						

Representatives sequences of *Bactrocera caudata* haplotypes found in this study deposited at GenBank are bolded.

The genomic DNAs of *Bactrocera* used in this study were isolated from samples preserved in absolute ethanol using i-genomic CTB DNA Extraction Mini Kit (iNtRON Biotechnology, Inc, Korea) as in Lim *et al*. [7]. The PCR primers and PCR amplifications for mitochondrial encoded markers, 16S[primers: (16S-F) LR-J-13756 5’-TAGTTTTTTTAGAAATAAATTTAATTTA-3’ and (16S-R) LR-N-13308 5’-GCCTTCAATTAAAAGACTAA-3’ [5] and COI [primers: UEA7-5’-TACAGTTGGAATAGACGTTGATAC-3’ and UEA10-5’-CCAATGCACTAATCTGCCATATTA-3’ [8]] were as described in Lim *et al*. [7], and for the nuclear encoded marker 28S [primers: 28sf, 5’-AAGGTAGCCAAATGCCTCATC-3’; 28sr, 5’-AGTAGGGTAAAACTAACCT-3’ [9]] as described in Lim *et al*. [10]. The primers for ITS-2 were ITS2F.dip-t: 5'- TGTAAAACGACGGCCAGTTGCTTGGACTACATATGGTTGA-3' and ITS2R.dip-t: 5'- CAGGAAACAGCTATGACGTAGTCCCATATGAGTTGAGGTT -3'[11]. The COII primers were: C2KD-F: 5'-CAAATTCGAATTTTAGTAACAGC-3' and C2KD-R: 5'- TTAGTTTGACAWACTAATGTTAT-3' [5]. The PCR parameters for amplification were as in Lim *et al*. [7,10] with the annealing temperature of 55°C. The same PCR primers were used for the DNA sequencing.

### Data Analysis

The sequences generated from this study and also other sequences of *B*. *caudata* from the GenBank (Table 1) were used for data analyses. The sequence data were edited and assembled using ChromasPro v.1.5 (Technelysium Pty Ltd., Australia) software followed by alignment with ClustalX program [12]. The analyzed data were trimmed using BioEdit v.7.0.5.3 [13].

Kakusan v.3 [14] was used to determine the best-fit nucleotide substitution models for maximum likelihood (ML) and Bayesian (BI) analyses, selected using the corrected Akaike Information Criterion [15] and the Bayesian Information Criterion [16], respectively. Phylograms were constructed using TreeFinder [17] prior to the annotations of bootstrap values (BP) generated via 1,000 ML bootstrap replicates. Bayesian analyses were conducted using the Markov Chain Monte Carlo (MCMC) method via Mr. Bayes v.3.1.2 [18]. Two independent runs of 2x10^6^ generations with four chains were performed, with trees sampled every 200^th^ generation. Likelihood values for all post-analysis trees and parameters were evaluated for convergence and burn-in using the “sump” command in MrBayes and the computer program Tracer v.1.5 (http://tree.bio.ed.ac.uk/software/tracer/). The first 200 trees from each run were discarded as burn-in (where the likelihood values were stabilized prior to the burn-in), and the remaining trees were used for the construction of a 50% majority-rule consensus tree. The effective sample size for COI+COII+16S was 1374.35 and for COI+COII+16S+28S+ITS-2 was 9268.35.

### Haplotype Network Reconstruction and Genetic Divergence

The median joining (MJ) network [19] was used to estimate the genealogical relationships of the haplotypes. The MJ network was calculated using NETWORK v4.6.1.0 (http://www.fluxus-engineering.com). PAUP* 4.0b10 software [20] was used to access the level of variation in the COI, COII and 16S rDNA among the selected samples of different taxa based on uncorrected (p) pairwise genetic distances.

## Results

### Haplotype Diversity

**Fig 1:**
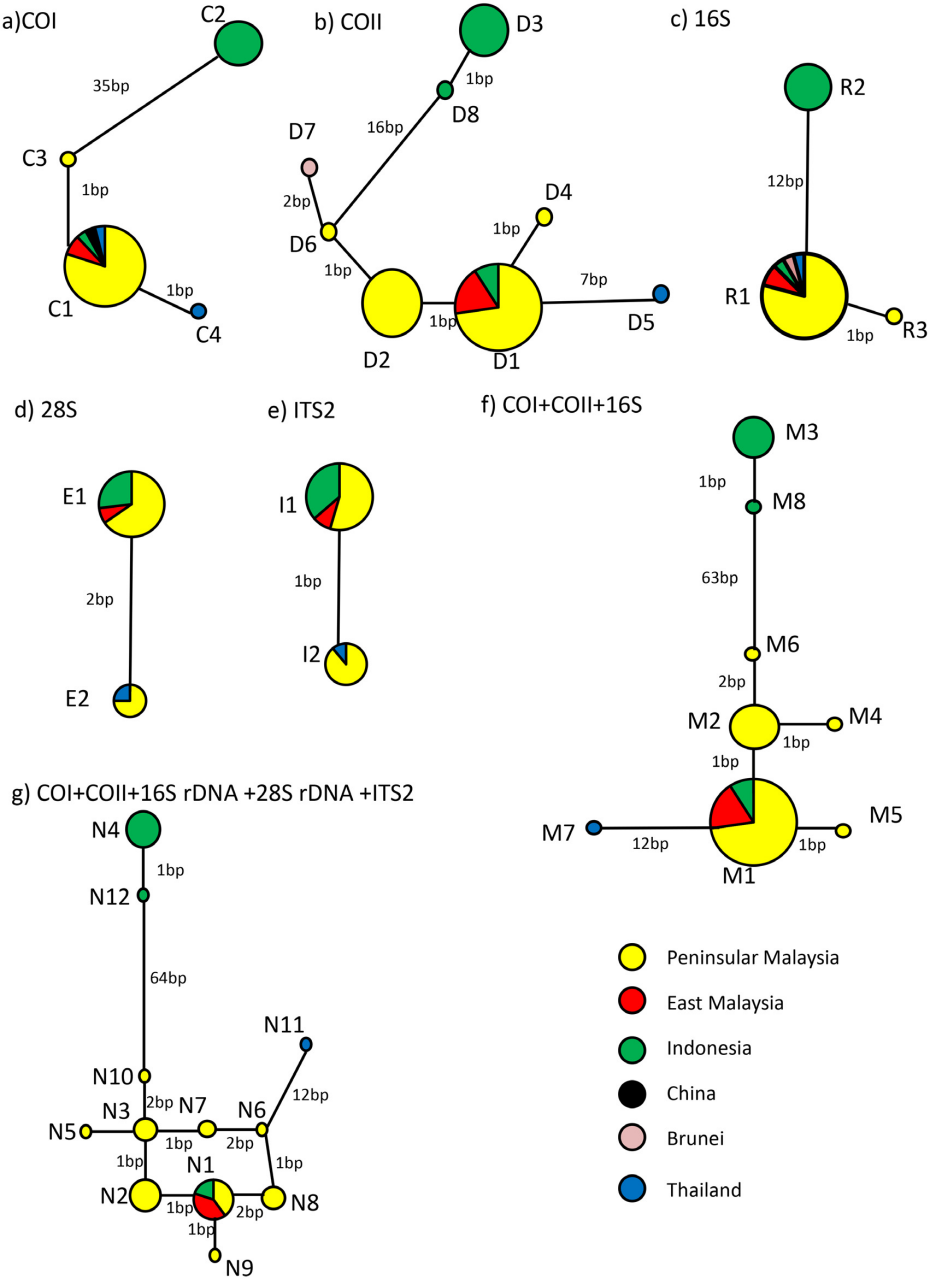
Median joining network of *Bactrocera caudata* of COI, COII, 16S rDNA, 28S rDNA, ITS-2, and concatenated COI+COII+16S and COI+COII+16S rDNA+28S+ITS-2 nucleotide sequences. Circle represents haplotype and sizes are relative to the number of individuals sharing the specific haplotype.

**Fig 2:**
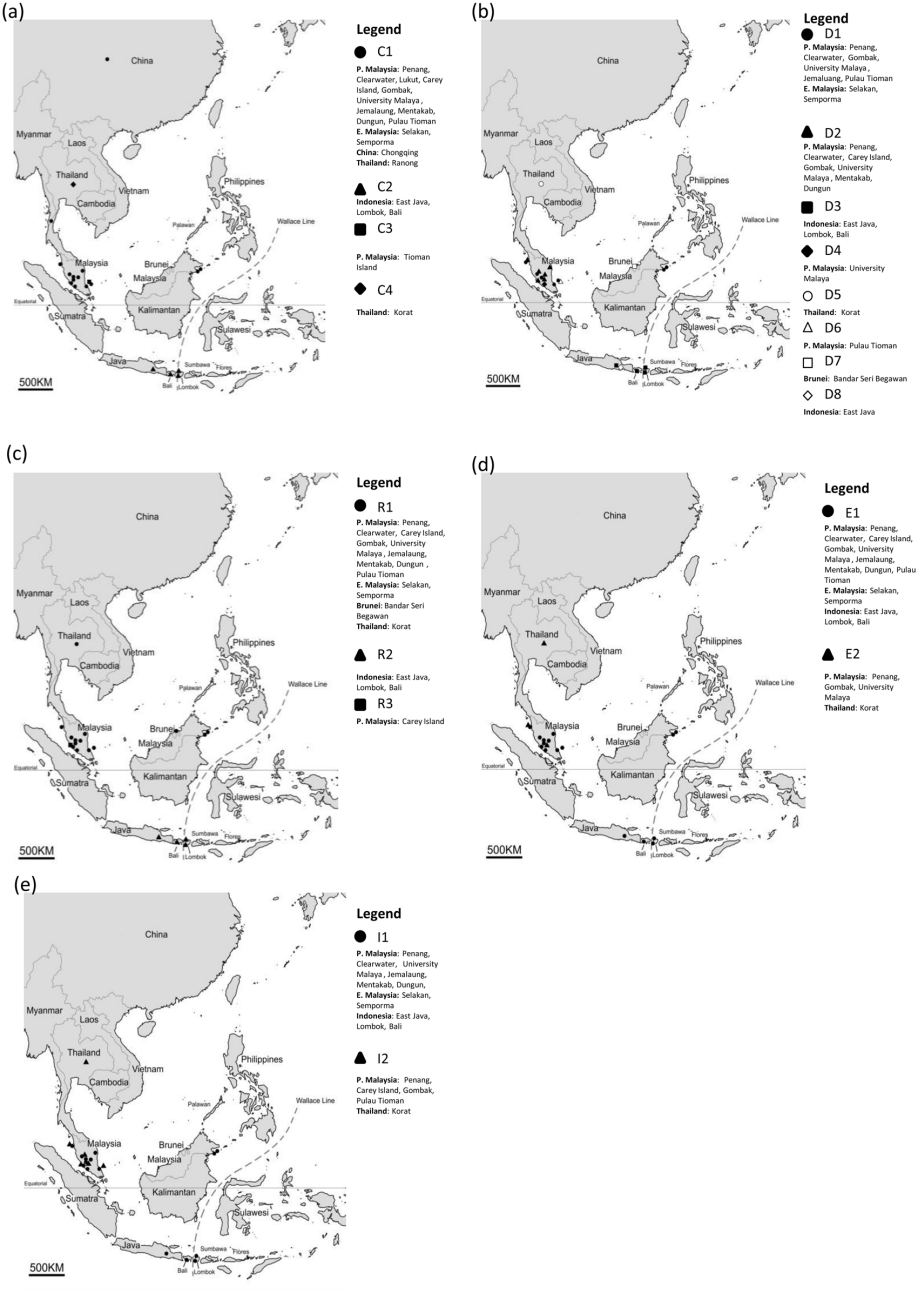
Sampling locations and corresponding haplotypes of *Bactrocera caudata* used in this study. (a) COI; (b) COII; (c) 16S rDNA (d) 28S rDNA and (e) ITS2.

The number of haplotypes based on the median-joining (MJ) network of the individual and concatenated aligned nucleotide sequences (outgroup was not included) is shown in Fig 1 and summarized in Table 1. The maps corresponding to the locations and hoplotypes are shown in Fig 2. The DNA variation sites for individual markers are shown in Tables A-E in S1 File. The 28S rRNA and ITS-2 genes were least variable, with two haplotypes each. Only one ITS-2 haplotype was found in the Indonesian taxa. Two 28S haplotypes were present in the populations of Peninsular Malaysia, East Malaysia, Indonesia and Thailand, with 2-bp difference.

Of the four COI haplotypes, C1 was the most common among the populations of Peninsular Malaysia and East Malaysia. Haplotype C2 occurred only in Indonesia, C3 in Pulau Tioman (Peninsular Malaysia) and C4 in Thailand. The largest difference of 37 bp was found between haplotypes C2 (Indonesia) and C4 (Thailand). Of the eight COII haplotypes, D1 was the most common. The Indonesian taxa were characterized by haplotypes D3 and D8. The largest difference of 28 bp was between haplotypes D3 (Indonesia) and D5 (Thailand). Three 16S haplotypes were present, with haplotype R2 present only in Indonesia. The largest difference of 13 bp was between haplotypes R2 (Indonesia) and R3 (Peninsular Malaysia).

The concatenated COI+COII+16S rDNA nucleotide sequences yielded eight haplotypes, with haplotypes M3 and M8 present only in Indonesia. The largest difference of 79 bp was between haplotypes M3 (Indonesia) and M8 (Thailand). A great diversity of haplotypes (12) was evident in the concatenated COI+COII+16S rDNA+28S rDNA+ITS-2 nucleotide sequences. The Indonesian taxa were characterized by haplotypes N4 and N12, with N4 having 82-bp difference from N11 (Thailand).

### Phylogenetic Relationships

The total length of the aligned sequences of 28S rRNA, ITS-2, COI, COII, 16S rRNA, 28S+ITS-2+COI+COII+16S rRNA, and COI+COII+16S rRNA genes as well as the selected models used for ML and BI analyses are summarized in Table 2.

**Table 2:** Information of the aligned sequences of 28S rRNA, ITS-2, COI, COII, 16S rRNA, COI+COII_16S and 28S+ITS-2+COI+COII+16S genes of *Bactrocera caudata*.

Data set	No. taxa	Total length	Model selected based on AIC	Model selected based on BIC
28S rRNA	33	818	HKY85+Homogeneous	HKY85+Homogeneous
ITS-2	32	455	HKY85+Homogeneous	HKY85+Homogeneous
COI	35	637	HKY+Gamma	J2+Gamma
COII	33	401	TN93+Gamma	HKY85+Homogeneous
16S rRNA	33	438	TIM+Gamma	HKY85+Gamma
COI+COII+16S	32	1476	TN+Gamma	TN93+Gamma
COI+COII+16S+28S+ITS-2	32	2749	J2+Gamma	TN93+Gamma

AIC, Akaike Information Criterion; BIC, Bayesian Information Criterion.

**Fig 3:**
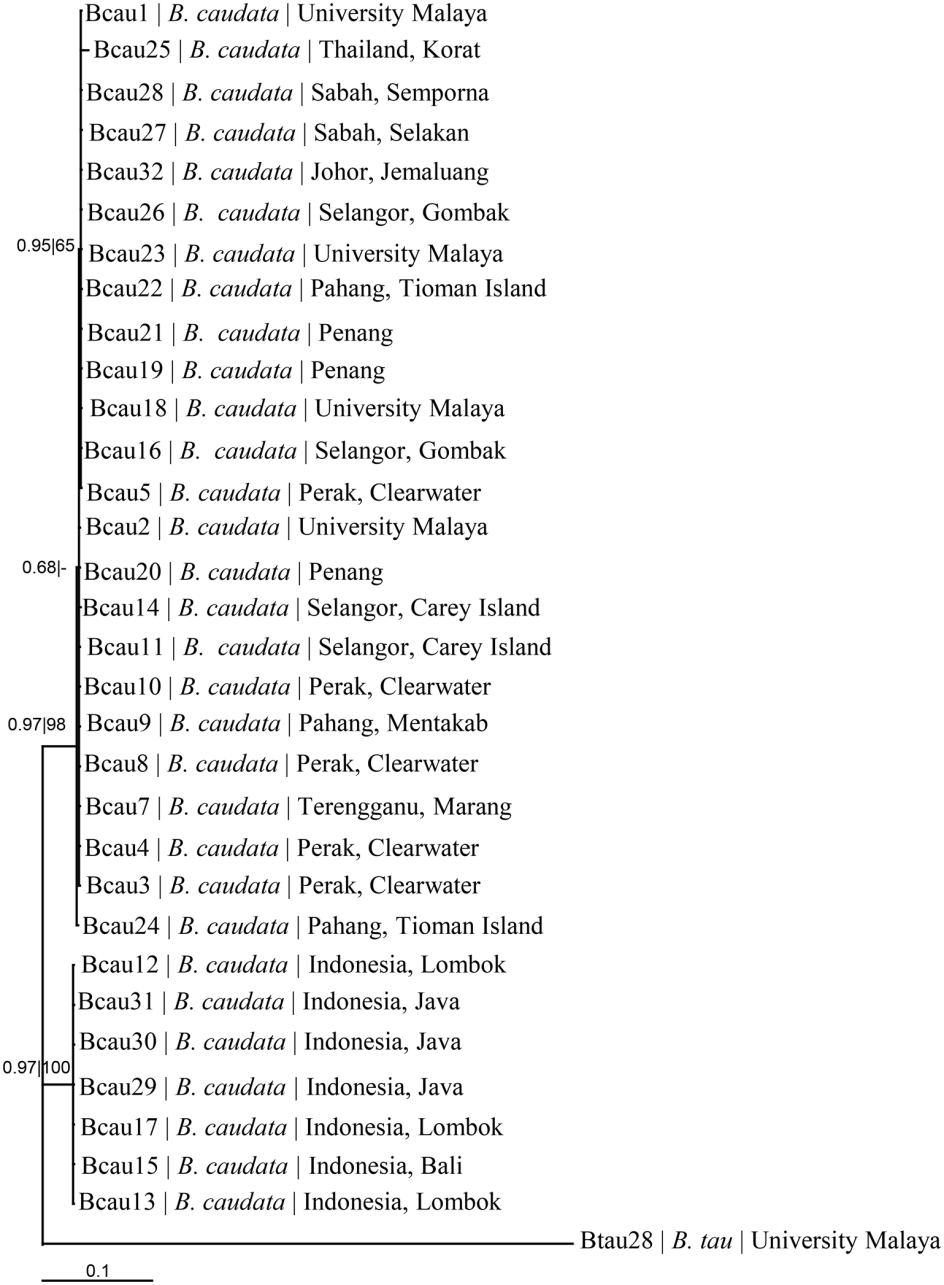
Phylogeny based on COI+COII+16S nucleotide sequences. Numeric values at the nodes are Bayesian posterior probabilities | ML bootstrap.

**Fig 4:**
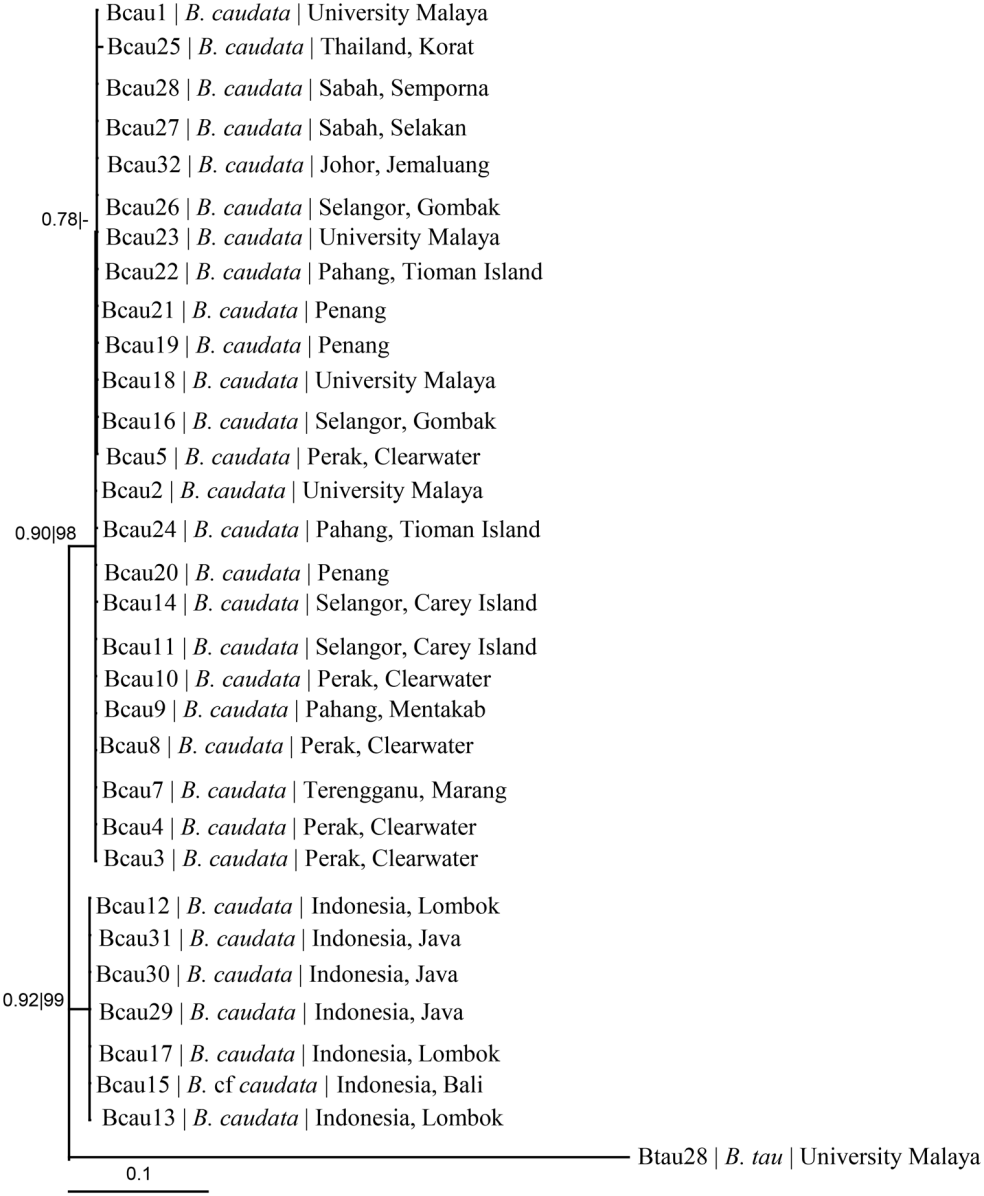
Phylogeny based on COI+COII+16S+28S+ITS-2 nucleotide sequences. Numeric values at the nodes are Bayesian posterior probabilities | ML bootstrap.

The support values and the grouping of the taxa of *B*. *caudata* varied from one marker to the others. The nucleotide sequences of the slower-evolving nuclear ITS-2 and 28S rRNA genes could not differentiate unequivocally the taxa of *B*. *caudata* from northern (Malaysia, Thailand) and southern (Indonesia) hemispheres (Table 1). The mitochondrial COI, COII and 16S rDNA nucleotide sequences unequivocally distinguish the taxa from these hemispheres, as also reflected by the concatenated COI+COII+16S rDNA (Fig 3) and 28S rDNA+ITS-2+COI+COII+16S rDNA (Fig 4) nucleotide sequences.

### Genetic Divergence

The uncorrected ‘p’ distances between different taxa of *B*. *caudata* based on COI, COII, 16S rDNA, 28S rDNA, ITS-2 (see Tables F-J in S1 File for the “p” distance for individual markers) and concatenated COI+COII+16S and COI+COII+16S+28S+ITS-2 nucleotide sequences are summarized in Table 3. Excepting 28S rRNA and ITS-2 genes, the uncorrected 'p' distances between the southern and northern hemisphere taxa for COI, COII, 16S and the concatenated sequences were several times higher than intra hemisphere values.

**Table 3:** Percentage of uncorrected “p” distance matrix between representative *Bactocera caudata* from various geographical locations in northern and southern hemispheres based on COI, COII, 16SrDNA, 28S rDNA, ITS-2 and concatenated COI+COII+16S and COI+COII+16S+28S+ITS-2 nucleotide sequences.

Location	COI	COII	16S	28S	ITS-2	Concatenated COI+COII+16S	Concatenated COI+COII+16S+28S+ITS-2
Intra Northern Hemisphere	0.00–0.94%	0.00–1.66%	0.00–0.23%	0.00%	0.00%	0.00–0.77%	0.00–0.41%
Intra Southern Hemisphere	0.00%	0.00%	0.00%	0.00%	0.00%	0.00%	0.00%
Northern vs SouthernHemisphere	5.49–6.12%	4.12–5.49%	2.76–2.99%	0.00%	0.00%	4.46–4.53%	2.34–2.69%

Based on concatenated COI+COII+16S nucleotide sequences, the Thailand (Korat) taxon differed from the other northern hemisphere (Malaysia) taxa with uncorrected 'p' distances of 0.56–0.77%; the 'p' distance for the Malaysian taxa was 0.00–0.21%. The uncorrected 'p' distance between the southern hemisphere (Indonesia) and Malaysian taxa was 4.46–4.53%, and 4.94% between Indonesian and Thailand taxa.

For concatenated COI+COII+16S+28S+ITS-2 nucleotide sequences, the uncorrected 'p' distance between Indonesian and Malaysian taxa was 2.34–2.50% and between Indonesian and Thailand taxa 2.69%. The uncorrected 'p' distance for Malaysian taxa was 0.00–0.19%, and between Malaysian and Thailand taxa 0.34–0.41%.

## Discussion

In the present study, the taxa of *B*. *caudata* from the northern (Malaysia, Thailand) and southern (Indonesia) hemispheres form clearly two distinct genetic lineages (Fig 3).

The Tephritid genus *Bactrocera* is represented by several species complexes, especially in the subgenus *Bactrocera* [21]. An example in the subgenus *Zeugodacus* is the *B*. *tau* species complex represented by some seven cryptic species [22, 23]. Morphological characters have proven to be problematic in identifying these sibling/cryptic species. Currently, molecular sequence data are used for determining the systematic status and phylogenetic relationship at various taxonomic levels, e.g. *Bactrocera dorsalis* species complex [24], molecular phylogeny of Dacini tribe [25], and higher phylogeny of frugivorous flies [26]. The mitochondrial COI, COII and 16S rRNA genes have been commonly used to study the phylogenetics of *Bactrocera* species [5,7,27–31]. COI sequences revealed the occurrence of eight species of the *Bactrocera tau* complex [32]. Species in the *B*. *tau* complex showed a sequence divergence of 0.06 to 28%, and the sequence divergence was up to 29% between the complex and its tephritid outgroups, *B*. *dorsalis* and *C*. *capitata* [32].

In an earlier study we showed that *B*. *caudata* of Malaysia-Thailand-China in the northern hemisphere was genetically distinct from *B*. *caudata* of Bali-Lombok in the southern hemisphere [7]. It was not clear then which was the nominal taxon as the study did not include the taxon from Java, the type locality. In the present study, the taxon from Malang, Java was genetically similar to those of Bali and Lombok of Indonesia in the southern hemisphere. The occurrence of *B*. *caudata* in Lombok may be the result of introduction from Java and/or Bali.

The occurrence of two distinct genetic lineages of the taxa attributed to *B*. *caudata* based on morphology (possession of three yellow vittae on the thorax, a transverse black band across the furrow of the face, two pairs of scutellar bristles and a slightly enlarged costal band at the apex of the wing) [1,2] indicates that the taxa of northern (Malaysia and Thailand) and southern (Indonesia) hemispheres may be members of a species complex. In that case, in accordance with the type locality the Indonesian taxa belong to the nominal species. Thus the taxa from the northern hemisphere, if they were to constitute a cryptic species of the *B*. *caudata* species complex based on molecular data, need to be formally described as a new species.

In the present study, based on concatenated COI+COII+16S rDNA nucleotide sequences the Thailand and Malaysian taxa of *B*. *caudata* in the northern hemisphere had an uncorrected 'p' distance of 0.56–0.77%, compared to 'p' = 0.00–0.21% for the Malaysian taxa. The distinct difference is also reflected by the concatenated COI+COII+16S+28S+ITS-2 nucleotide sequences. It indicates the occurrence of distinct genetic structure and phylogeographic pattern of the *B*. *caudata* populations in the northern hemisphere.

## Supporting Information

S1 File Supporting tables. Table A in S1 File. Variation sites in DNA sequences for different haplotype of mitochondrial COI of *Bactrocera* species from various localities. Table B in S1 File. Variation sites in DNA sequences for different haplotype of mitochondrial COII of *Bactrocera* species from various localities. Table C in S1 File. Variation sites in DNA sequences of *Bactrocera* species for mitochondrial 16S rDNA from various localities. (Source: Lim et al. 2012). Table D in S1 File. Variation sites in DNA sequences of *Bactrocera* species for 28S rDNA from various localities. Table E is S1 File. Variation sites in DNA sequences of *Bactrocera* species for ITS-2 from various localities. Table F is S1 File. Percentage of uncorrected “p” distance matrix of *Bactocera caudata* from various geographical locations in northern and southern hemispheres based on COI. Table G in S1 File. Percentage of uncorrected “p” distance matrix of *Bactocera caudata* from various geographical locations in northern and southern hemispheres based on COII. Table H in S1 File. Percentage of uncorrected “p” distance matrix of *Bactocera caudata* from various geographical locations in northern and southern hemispheres based on 16SrDNA. Table I in S1 File. Percentage of uncorrected “p” distance matrix of *Bactocera caudata* from various geographical locations in northern and southern hemispheres based on 28S rDNA. Table J in S1 File. Percentage of uncorrected “p” distance matrix of *Bactocera caudata* from various geographical locations in northern and southern hemispheres based on ITS-2.
(DOCX)
