# Supplementary material for: Multigene Phylogeography of Bactrocera caudata (Insecta: Tephritidae): Distinct Genetic Lineages in Northern and Southern Hemispheres
Source: PLoS One. 2015 Jun 19;10(6):e0129455. doi: 10.1371/journal.pone.0129455 (PMC4474862; doi:10.1371/journal.pone.0129455)
Supplement: S1 File — Table A in S1 File. Variation sites in DNA sequences for different haplotype of mitochondrial COI of Bactrocera species from various localities. Table B in S1 File. Variation sites in DNA sequences for different haplotype of mitochondrial COII of Bactrocera species from various localities. Table C in S1 File. Variation sites in DNA sequences of Bactrocera species for mitochondrial 16S rDNA from various localities. (Source: Lim et al. 2012). Table D in S1 File. Variation sites in DNA sequences of Bactrocera species for 28S rDNA from various localities. Table E is S1 File. Variation sites in DNA sequences of Bactrocera species for ITS-2 from various localities. Table F is S1 File. Percentage of uncorrected “p” distance matrix of Bactocera caudata from various geographical locations in northern and southern hemispheres based on COI. Table G in S1 File. Percentage of uncorrected “p” distance matrix of Bactocera caudata from various geographical locations in northern and southern hemispheres based on COII. Table H in S1 File. Percentage of uncorrected “p” distance matrix of Bactocera caudata from various geographical locations in northern and southern hemispheres based on 16SrDNA. Table I in S1 File. Percentage of uncorrected “p” distance matrix of Bactocera caudata from various geographical locations in northern and southern hemispheres based on 28S rDNA. Table J in S1 File. Percentage of uncorrected “p” distance matrix of Bactocera caudata from various geographical locations in northern and southern hemispheres based on ITS-2. (DOCX) [file pone.0129455.s001.docx]

Table A. Variation sites in DNA sequences for different haplotype of mitochondrial COI of *Bactrocera* species from various localities.

| **Variation sites in DNA sequences** | **Haplotypes** | | | | **Variation sites in DNA sequences** | **Haplotypes** | | | | **Variation sites in DNA sequences** | **Haplotypes** | | | |
| --- | --- | --- | --- | --- | --- | --- | --- | --- | --- | --- | --- | --- | --- | --- |
|  | **C1** | **C2** | **C3** | **C4** |  | **C1** | **C2** | **C3** | **C4** |  | **C1** | **C2** | **C3** | **C4** |
| **9** | T | C | T | T | **180** | T | C | T | T | **481** | T | C | T | T |
| **84** | T | A | T | T | **216** | C | A | C | C | **483** | A | T | A | A |
| **96** | T | C | T | T | **234** | C | C | C | T | **489** | C | T | C | C |
| **99** | C | T | C | C | **288** | T | C | T | T | **499** | T | C | T | T |
| **102** | T | C | T | T | **321** | A | G | A | A | **546** | T | C | T | T |
| **108** | T | A | T | T | **325** | T | C | T | T | **549** | C | T | T | T |
| **117** | A | G | A | A | **366** | C | T | C | C | **588** | C | T | C | C |
| **120** | C | A | C | C | **372** | C | T | C | C | **594** | T | G | T | T |
| **129** | T | C | T | T | **399** | A | G | A | A | **600** | A | C | A | A |
| **136** | C | T | C | C | **417** | T | C | T | C | **606** | C | T | C | C |
| **147** | A | A | A | G | **429** | C | C | C | T | **615** | T | A | T | T |
| **153** | A | T | A | A | **438** | T | C | T | T | **619** | C | T | C | C |
| **168** | A | G | A | A | **450** | C | T | T | C |  |  |  |  |  |

Table B. Variation sites in DNA sequences for different haplotype of mitochondrial COII of *Bactrocera* species from various localities.

| **Variation sites in DNA sequences** | **Haplotypes** | | | | | | | | **Variation sites in DNA sequences** | **Haplotypes** | | | | | | | | | | | | | |
| --- | --- | --- | --- | --- | --- | --- | --- | --- | --- | --- | --- | --- | --- | --- | --- | --- | --- | --- | --- | --- | --- | --- | --- |
|  | **D1** | **D2** | **D3** | **D4** | **D5** | **D6** | **D7** | **D8** |  | **D1** | **D2** | | **D3** | | **D4** | | **D5** | | **D6** | | **D7** | | **D8** |
| **12** | A | T | T | A | **A** | **T** | **T** | **T** | **200** | C | | C | | T | | C | | C | | C | | C | T |
| **13** | - | - | - | A | **-** | **-** | **-** | **-** | **203** | A | | A | | T | | A | | A | | A | | A | T |
| **38** | T | T | C | T | **T** | **T** | **T** | **C** | **209** | C | | C | | T | | C | | C | | C | | C | T |
| **44** | T | T | C | T | **T** | **T** | **T** | **C** | **232** | G | | G | | A | | G | | G | | G | | G | A |
| **51** | G | G | A | G | **G** | **G** | **G** | **A** | **277** | C | | C | | TT | | C | | C | | C | | C | T |
| **53** | T | T | C | T | **T** | **T** | **T** | **C** | **294** | T | | T | | C | | T | | T | | T | | T | C |
| **83** | T | T | C | T | **T** | **T** | **T** | **C** | **295** | C | | C | | T | | C | | C | | C | | C | T |
| **119** | C | C | T | C | **C** | **C** | **C** | **T** | **324** | G | | G | | G | | G | | A | | G | | G | G |
| **122** | T | T | C | T | **T** | **C** | **C** | **C** | **331** | - | | - | | A | | - | | A | | - | | - | A |
| **158** | C | C | C | C | **T** | **C** | **C** | **C** | **332** | - | | - | | A | | - | | A | | - | | - | - |
| **161** | A | A | A | A | **G** | **A** | **A** | **A** | **342** | - | | - | | - | | - | | G | | - | | - | - |
| **173** | C | C | T | C | **C** | **C** | **C** | **T** | **343** | - | | - | | - | | - | | T | | - | | - | - |
| **185** | T | T | C | T | **T** | **T** | **T** | **C** |  |  | |  | |  | |  | |  | |  | |  |  |

Table C. Variation sites in DNA sequences of *Bactrocera* species for mitochondrial 16S rDNA from various localities. (Source: Lim et al. 2012)

| **Variation sites in DNA sequences** | **Haplotypes** | | | **Variation sites in DNA sequences** | **Haplotypes** | | |
| --- | --- | --- | --- | --- | --- | --- | --- |
|  | **R1** | **R2** | **R3** |  | **R1** | **R2** | **R3** |
| **23** | C | A | C | **213** | A | A | G |
| **30** | A | A | G | **260** | G | G | T |
| **151** | G | G | A | **325** | C | C | A |
| **160** | C | C | T | **326** | T | T | C |
| **161** | A | A | C | **398** | C | C | T |
| **187** | C | C | T | **420** | T | T | C |
| **206** | A | A | G |  |  |  |  |

Table D. Variation sites in DNA sequences of *Bactrocera* species for 28S rDNA from various localities.

| **Variation site on DNA sequence** | **Haplotypes** | |
| --- | --- | --- |
|  | **E1** | **E2** |
| **801** | T | - |
| **807** | G | - |

Table E. Variation sites in DNA sequences of *Bactrocera* species for ITS-2 from various localities.

| **Variation site on DNA sequence** | **Haplotypes** | |
| --- | --- | --- |
|  | **I1** | **I2** |
| **324** | A | G |

Table F. Percentage of uncorrected “p” distance matrix of *Bactocera caudata* from various geographical locations in northern and southern hemispheres based on COI.

|  | 1 | 2 | 3 | 4 | 5 | 6 | 7 | 8 | 9 | 10 | 11 | 12 | 13 | 14 | 15 | 16 | 17 | 18 | 19 | 20 | 21 | 22 | 23 | 24 | 25 | 26 | 27 | 28 | 29 | 30 | 31 | 32 | 33 | 34 | 35 |
| --- | --- | --- | --- | --- | --- | --- | --- | --- | --- | --- | --- | --- | --- | --- | --- | --- | --- | --- | --- | --- | --- | --- | --- | --- | --- | --- | --- | --- | --- | --- | --- | --- | --- | --- | --- |
| JN542419 \| *B. caudata* \| Bali, Indonesia | - |  |  |  |  |  |  |  |  |  |  |  |  |  |  |  |  |  |  |  |  |  |  |  |  |  |  |  |  |  |  |  |  |  |  |
| JN542418 \| *B. caudata* \| Lombok, Indonesia | 0.00 | - |  |  |  |  |  |  |  |  |  |  |  |  |  |  |  |  |  |  |  |  |  |  |  |  |  |  |  |  |  |  |  |  |  |
| Bcau29 \| *B. caudata* I Java, Indonesia | 0.00 | 0.00 | - |  |  |  |  |  |  |  |  |  |  |  |  |  |  |  |  |  |  |  |  |  |  |  |  |  |  |  |  |  |  |  |  |
| Bcau30 \| *B. caudata* \| Java, Indonesia | 0.00 | 0.00 | 0.00 | - |  |  |  |  |  |  |  |  |  |  |  |  |  |  |  |  |  |  |  |  |  |  |  |  |  |  |  |  |  |  |  |
| Bcau13 \| *B. caudata* \| Lombok, Indonesia | 0.00 | 0.00 | 0.00 | 0.00 | - |  |  |  |  |  |  |  |  |  |  |  |  |  |  |  |  |  |  |  |  |  |  |  |  |  |  |  |  |  |  |
| Bcau31 \| *B. caudata* \| Java, Indonesia | 0.00 | 0.00 | 0.00 | 0.00 | 0.00 | - |  |  |  |  |  |  |  |  |  |  |  |  |  |  |  |  |  |  |  |  |  |  |  |  |  |  |  |  |  |
| Bcau17 \| *B. caudata* \| Lombok, Indonesia | 0.00 | 0.00 | 0.00 | 0.00 | 0.00 | 0.00 | - |  |  |  |  |  |  |  |  |  |  |  |  |  |  |  |  |  |  |  |  |  |  |  |  |  |  |  |  |
| JN542417 \| *B. caudata* \| University Malaya, P. Malaysia | 5.65 | 5.65 | 5.65 | 5.65 | 5.65 | 5.65 | 5.65 | - |  |  |  |  |  |  |  |  |  |  |  |  |  |  |  |  |  |  |  |  |  |  |  |  |  |  |  |
| JN542416 \| *B. caudata* \| Carey Island, P. Malaysia | 5.65 | 5.65 | 5.65 | 5.65 | 5.65 | 5.65 | 5.65 | 0.00 | - |  |  |  |  |  |  |  |  |  |  |  |  |  |  |  |  |  |  |  |  |  |  |  |  |  |  |
| AF423109 \| *B. caudata* \| ‘Thailand’ | 5.65 | 5.65 | 5.65 | 5.65 | 5.65 | 5.65 | 5.65 | 0.00 | 0.00 | - |  |  |  |  |  |  |  |  |  |  |  |  |  |  |  |  |  |  |  |  |  |  |  |  |  |
| Bcau19 \| *B. caudata* \| Penang | 5.65 | 5.65 | 5.65 | 5.65 | 5.65 | 5.65 | 5.65 | 0.00 | 0.00 | 0.00 | - |  |  |  |  |  |  |  |  |  |  |  |  |  |  |  |  |  |  |  |  |  |  |  |  |
| Bcau9 \| *B. caudata* \| Mentakab, P. Malaysia | 5.65 | 5.65 | 5.65 | 5.65 | 5.65 | 5.65 | 5.65 | 0.00 | 0.00 | 0.00 | 0.00 | - |  |  |  |  |  |  |  |  |  |  |  |  |  |  |  |  |  |  |  |  |  |  |  |
| Bcau11 \| *B. caudata* \| Carey Island, P. Malaysia | 5.65 | 5.65 | 5.65 | 5.65 | 5.65 | 5.65 | 5.65 | 0.00 | 0.00 | 0.00 | 0.00 | 0.00 | - |  |  |  |  |  |  |  |  |  |  |  |  |  |  |  |  |  |  |  |  |  |  |
| Bcau7 \| *B. caudata* \| Terengganu, Marang | 5.65 | 5.65 | 5.65 | 5.65 | 5.65 | 5.65 | 5.65 | 0.00 | 0.00 | 0.00 | 0.00 | 0.00 | 0.00 | - |  |  |  |  |  |  |  |  |  |  |  |  |  |  |  |  |  |  |  |  |  |
| Bcau5 \| *B. caudata* \| Clearwater, , P. Malaysia | 5.65 | 5.65 | 5.65 | 5.65 | 5.65 | 5.65 | 5.65 | 0.00 | 0.00 | 0.00 | 0.00 | 0.00 | 0.00 | 0.00 | - |  |  |  |  |  |  |  |  |  |  |  |  |  |  |  |  |  |  |  |  |
| Bcau8 \| *B. caudata* \| Clearwater, P. Malaysia | 5.65 | 5.65 | 5.65 | 5.65 | 5.65 | 5.65 | 5.65 | 0.00 | 0.00 | 0.00 | 0.00 | 0.00 | 0.00 | 0.00 | 0.00 | - |  |  |  |  |  |  |  |  |  |  |  |  |  |  |  |  |  |  |  |
| Bcau4 \| *B. caudata* \| Perak, Clearwater | 5.65 | 5.65 | 5.65 | 5.65 | 5.65 | 5.65 | 5.65 | 0.00 | 0.00 | 0.00 | 0.00 | 0.00 | 0.00 | 0.00 | 0.00 | 0.00 | - |  |  |  |  |  |  |  |  |  |  |  |  |  |  |  |  |  |  |
| Bcau23 \| *B. caudata* \| University Malaya, P. Malaysia | 5.65 | 5.65 | 5.65 | 5.65 | 5.65 | 5.65 | 5.65 | 0.00 | 0.00 | 0.00 | 0.00 | 0.00 | 0.00 | 0.00 | 0.00 | 0.00 | 0.00 | - |  |  |  |  |  |  |  |  |  |  |  |  |  |  |  |  |  |
| Bcau26 \| *B. caudata* \| Gombak, P. Malaysia | 5.65 | 5.65 | 5.65 | 5.65 | 5.65 | 5.65 | 5.65 | 0.00 | 0.00 | 0.00 | 0.00 | 0.00 | 0.00 | 0.00 | 0.00 | 0.00 | 0.00 | 0.00 | - |  |  |  |  |  |  |  |  |  |  |  |  |  |  |  |  |
| Bcau28 \| *B. caudata* \| Sabah, Semporna | 5.65 | 5.65 | 5.65 | 5.65 | 5.65 | 5.65 | 5.65 | 0.00 | 0.00 | 0.00 | 0.00 | 0.00 | 0.00 | 0.00 | 0.00 | 0.00 | 0.00 | 0.00 | 0.00 | - |  |  |  |  |  |  |  |  |  |  |  |  |  |  |  |
| Bcau3 \| *B. caudata* \| Clearwater, P. Malaysia | 5.65 | 5.65 | 5.65 | 5.65 | 5.65 | 5.65 | 5.65 | 0.00 | 0.00 | 0.00 | 0.00 | 0.00 | 0.00 | 0.00 | 0.00 | 0.00 | 0.00 | 0.00 | 0.00 | 0.00 | - |  |  |  |  |  |  |  |  |  |  |  |  |  |  |
| Bcau10 \| *B. caudata* \| Clearwater, , P. Malaysia | 5.65 | 5.65 | 5.65 | 5.65 | 5.65 | 5.65 | 5.65 | 0.00 | 0.00 | 0.00 | 0.00 | 0.00 | 0.00 | 0.00 | 0.00 | 0.00 | 0.00 | 0.00 | 0.00 | 0.00 | 0.00 | - |  |  |  |  |  |  |  |  |  |  |  |  |  |
| Bcau27 \| *B. caudata* \| Selakan, Sabah, E. Malaysia | 5.65 | 5.65 | 5.65 | 5.65 | 5.65 | 5.65 | 5.65 | 0.00 | 0.00 | 0.00 | 0.00 | 0.00 | 0.00 | 0.00 | 0.00 | 0.00 | 0.00 | 0.00 | 0.00 | 0.00 | 0.00 | 0.00 | - |  |  |  |  |  |  |  |  |  |  |  |  |
| Bcau32 \| *B. caudata* \| Jemaluang, P. Malaysia | 5.65 | 5.65 | 5.65 | 5.65 | 5.65 | 5.65 | 5.65 | 0.00 | 0.00 | 0.00 | 0.00 | 0.00 | 0.00 | 0.00 | 0.00 | 0.00 | 0.00 | 0.00 | 0.00 | 0.00 | 0.00 | 0.00 | 0.00 | - |  |  |  |  |  |  |  |  |  |  |  |
| Bcau21 \| *B. caudata* \| Penang, P. Malaysia | 5.65 | 5.65 | 5.65 | 5.65 | 5.65 | 5.65 | 5.65 | 0.00 | 0.00 | 0.00 | 0.00 | 0.00 | 0.00 | 0.00 | 0.00 | 0.00 | 0.00 | 0.00 | 0.00 | 0.00 | 0.00 | 0.00 | 0.00 | 0.00 | - |  |  |  |  |  |  |  |  |  |  |
| Bcau20 \| *B. caudata* \| Penang, P. Malaysia | 5.65 | 5.65 | 5.65 | 5.65 | 5.65 | 5.65 | 5.65 | 0.00 | 0.00 | 0.00 | 0.00 | 0.00 | 0.00 | 0.00 | 0.00 | 0.00 | 0.00 | 0.00 | 0.00 | 0.00 | 0.00 | 0.00 | 0.00 | 0.00 | 0.00 | - |  |  |  |  |  |  |  |  |  |
| Bcau16 \| *B. caudata* \| Gombak, , P. Malaysia | 5.65 | 5.65 | 5.65 | 5.65 | 5.65 | 5.65 | 5.65 | 0.00 | 0.00 | 0.00 | 0.00 | 0.00 | 0.00 | 0.00 | 0.00 | 0.00 | 0.00 | 0.00 | 0.00 | 0.00 | 0.00 | 0.00 | 0.00 | 0.00 | 0.00 | 0.00 | - |  |  |  |  |  |  |  |  |
| Bcau18 \| *B. caudata* \| University Malaya, P. Malaysia | 5.65 | 5.65 | 5.65 | 5.65 | 5.65 | 5.65 | 5.65 | 0.00 | 0.00 | 0.00 | 0.00 | 0.00 | 0.00 | 0.00 | 0.00 | 0.00 | 0.00 | 0.00 | 0.00 | 0.00 | 0.00 | 0.00 | 0.00 | 0.00 | 0.00 | 0.00 | 0.00 | - |  |  |  |  |  |  |  |
| Bcau22 \| *B. caudata* \| Tioman Island, P. Malaysia | 5.65 | 5.65 | 5.65 | 5.65 | 5.65 | 5.65 | 5.65 | 0.00 | 0.00 | 0.00 | 0.00 | 0.00 | 0.00 | 0.00 | 0.00 | 0.00 | 0.00 | 0.00 | 0.00 | 0.00 | 0.00 | 0.00 | 0.00 | 0.00 | 0.00 | 0.00 | 0.00 | 0.00 | - |  |  |  |  |  |  |
| Bcau1 \| *B. caudata* \| University Malaya, P. Malaysia | 5.65 | 5.65 | 5.65 | 5.65 | 5.65 | 5.65 | 5.65 | 0.00 | 0.00 | 0.00 | 0.00 | 0.00 | 0.00 | 0.00 | 0.00 | 0.00 | 0.00 | 0.00 | 0.00 | 0.00 | 0.00 | 0.00 | 0.00 | 0.00 | 0.00 | 0.00 | 0.00 | 0.00 | 0.00 | - |  |  |  |  |  |
| FJ903493 \| *B. caudata* \| Malaysia | 5.65 | 5.65 | 5.65 | 5.65 | 5.65 | 5.65 | 5.65 | 0.00 | 0.00 | 0.00 | 0.00 | 0.00 | 0.00 | 0.00 | 0.00 | 0.00 | 0.00 | 0.00 | 0.00 | 0.00 | 0.00 | 0.00 | 0.00 | 0.00 | 0.00 | 0.00 | 0.00 | 0.00 | 0.00 | 0.00 | - |  |  |  |  |
| GQ458048 \| *B. caudata* \| Chongqing, China | 5.65 | 5.65 | 5.65 | 5.65 | 5.65 | 5.65 | 5.65 | 0.00 | 0.00 | 0.00 | 0.00 | 0.00 | 0.00 | 0.00 | 0.00 | 0.00 | 0.00 | 0.00 | 0.00 | 0.00 | 0.00 | 0.00 | 0.00 | 0.00 | 0.00 | 0.00 | 0.00 | 0.00 | 0.00 | 0.00 | 0.00 | - |  |  |  |
| Bcau24 \| *B. caudata* \| Tioman Island, P. Malaysia | 5.50 | 5.50 | 5.50 | 5.50 | 5.50 | 5.50 | 5.50 | 0.16 | 0.16 | 0.16 | 0.16 | 0.16 | 0.16 | 0.16 | 0.16 | 0.16 | 0.16 | 0.16 | 0.16 | 0.16 | 0.16 | 0.16 | 0.16 | 0.16 | 0.16 | 0.16 | 0.16 | 0.16 | 0.16 | 0.16 | 0.16 | 0.16 | - |  |  |
| Bcau25 \| *B. caudata* \| Korat, Thailand | 6.12 | 6.12 | 6.12 | 6.12 | 6.12 | 6.12 | 6.12 | 0.79 | 0.79 | 0.79 | 0.79 | 0.79 | 0.79 | 0.79 | 0.79 | 0.79 | 0.79 | 0.79 | 0.79 | 0.79 | 0.79 | 0.79 | 0.79 | 0.79 | 0.79 | 0.79 | 0.79 | 0.79 | 0.79 | 0.79 | 0.79 | 0.79 | 0.94 | - |  |

Table G. Percentage of uncorrected “p” distance matrix of *Bactocera caudata* from various geographical locations in northern and southern hemispheres based on COII.

|  | 1 | 2 | 3 | 4 | 5 | 6 | 7 | 8 | | 9 | | 10 | | 11 | | 12 | | 13 | | 14 | | 15 | | 16 | | 17 | | 18 | | 19 | | 20 | | 21 | | 22 | | 23 | | 24 | | 25 | | 26 | | 27 | | 28 | | 29 | | 30 | | 31 | | 32 | | 33 | |  |
| --- | --- | --- | --- | --- | --- | --- | --- | --- | --- | --- | --- | --- | --- | --- | --- | --- | --- | --- | --- | --- | --- | --- | --- | --- | --- | --- | --- | --- | --- | --- | --- | --- | --- | --- | --- | --- | --- | --- | --- | --- | --- | --- | --- | --- | --- | --- | --- | --- | --- | --- | --- | --- | --- | --- | --- | --- | --- | --- | --- | --- |
| Bcau13 \| *B. caudata* \| Lombok, Indonesia | - |  |  |  |  |  |  |  | |  | |  | |  | |  | |  | |  | |  | |  | |  | |  | |  | |  | |  | |  | |  | |  | |  | |  | |  | |  | |  | |  | |  | |  | |  | |  |
| Bcau15 \| *B. caudata* \| Bali, Indonesia | 0.00 | - |  |  |  |  |  |  | |  | |  | |  | |  | |  | |  | |  | |  | |  | |  | |  | |  | |  | |  | |  | |  | |  | |  | |  | |  | |  | |  | |  | |  | |  | |  |
| Bcau31 \| *B. caudata* \| Java, Indonesia | 0.00 | 0.00 | - |  |  |  |  |  | |  | |  | |  | |  | |  | |  | |  | |  | |  | |  | |  | |  | |  | |  | |  | |  | |  | |  | |  | |  | |  | |  | |  | |  | |  | |  |
| Bcau12 \| *B. caudata* \| Lombok Indonesia | 0.00 | 0.00 | 0.00 | - |  |  |  |  | |  | |  | |  | |  | |  | |  | |  | |  | |  | |  | |  | |  | |  | |  | |  | |  | |  | |  | |  | |  | |  | |  | |  | |  | |  | |  |
| Bcau29 \| *B. caudata \|* Java, Indonesia | 0.00 | 0.00 | 0.00 | 0.00 | - |  |  |  | |  | |  | |  | |  | |  | |  | |  | |  | |  | |  | |  | |  | |  | |  | |  | |  | |  | |  | |  | |  | |  | |  | |  | |  | |  | |  |
| Bcau17 \| *B. caudata* \| Lombok, Indonesia | 0.00 | 0.00 | 0.00 | 0.00 | 0.00 | - |  |  | |  | |  | |  | |  | |  | |  | |  | |  | |  | |  | |  | |  | |  | |  | |  | |  | |  | |  | |  | |  | |  | |  | |  | |  | |  | |  |
| Bcau30 \| *B. caudata* \| Java, Indonesia | 0.00 | 0.00 | 0.00 | 0.00 | 0.00 | 0.00 | - |  | |  | |  | |  | |  | |  | |  | |  | |  | |  | |  | |  | |  | |  | |  | |  | |  | |  | |  | |  | |  | |  | |  | |  | |  | |  | |  |
| Bcau23 \| *B. caudata* \| University Malaya, P. Malaysia | 4.66 | 4.66 | 4.66 | 4.66 | 4.66 | 4.66 | 4.67 | | - | |  | |  | |  | |  | |  | |  | |  | |  | |  | |  | |  | |  | |  | |  | |  | |  | |  | |  | |  | |  | |  | |  | |  | |  | |  | |
| Bcau22 \| *B. caudata* \| Tioman Island, P. Malaysia | 4.67 | 4.67 | 4.67 | 4.67 | 4.67 | 4.67 | 4.68 | | 0.00 | | - | |  | |  | |  | |  | |  | |  | |  | |  | |  | |  | |  | |  | |  | |  | |  | |  | |  | |  | |  | |  | |  | |  | |  | |  | |
| Bcau32 \| *B. caudata* \| Jemaluang, P. Malaysia | 4.67 | 4.67 | 4.67 | 4.67 | 4.67 | 4.67 | 4.68 | | 0.00 | | 0.00 | | - | |  | |  | |  | |  | |  | |  | |  | |  | |  | |  | |  | |  | |  | |  | |  | |  | |  | |  | |  | |  | |  | |  | |  | |
| Bcau5 \| *B. caudata* \| Clearwater, , P. Malaysia | 4.67 | 4.67 | 4.67 | 4.67 | 4.67 | 4.67 | 4.68 | | 0.00 | | 0.00 | | -- | | - | |  | |  | |  | |  | |  | |  | |  | |  | |  | |  | |  | |  | |  | |  | |  | |  | |  | |  | |  | |  | |  | |  | |
| Bcau1 \| *B. caudata* \| University Malaya, P. Malaysia | 4.67 | 4.67 | 4.67 | 4.67 | 4.67 | 4.67 | 4.68 | | 0.00 | | 0.00 | | 0.00 | | 0.00 | | - | |  | |  | |  | |  | |  | |  | |  | |  | |  | |  | |  | |  | |  | |  | |  | |  | |  | |  | |  | |  | |  | |
| Bcau21 \| *B. caudata* \| Penang, P. Malaysia | 4.67 | 4.67 | 4.67 | 4.67 | 4.67 | 4.67 | 4.68 | | 0.00 | | 0.00 | | 0.00 | | 0.00 | | 0.00 | | - | |  | |  | |  | |  | |  | |  | |  | |  | |  | |  | |  | |  | |  | |  | |  | |  | |  | |  | |  | |  | |
| Bcau16 \| *B. caudata* \| Gombak, P. Malaysia | 4.67 | 4.67 | 4.67 | 4.67 | 4.67 | 4.67 | 4.68 | | 0.00 | | 0.00 | | 0.00 | | 0.00 | | 0.00 | | 0.00 | | - | |  | |  | |  | |  | |  | |  | |  | |  | |  | |  | |  | |  | |  | |  | |  | |  | |  | |  | |  | |
| Bcau27 \| *B. caudata* \| Selakan, E. Malaysia | 4.67 | 4.67 | 4.67 | 4.67 | 4.67 | 4.67 | 4.68 | | 0.00 | | 0.00 | | 0.00 | | 0.00 | | 0.00 | | 0.00 | | 0.00 | | - | |  | |  | |  | |  | |  | |  | |  | |  | |  | |  | |  | |  | |  | |  | |  | |  | |  | |  | |
| Bcau26 \| *B. caudata* \| Gombak, P. Malaysia | 4.67 | 4.67 | 4.67 | 4.67 | 4.67 | 4.67 | 4.68 | | 0.00 | | 0.00 | | 0.00 | | 0.00 | | 0.00 | | 0.00 | | 0.00 | | 0.00 | | - | |  | |  | |  | |  | |  | |  | |  | |  | |  | |  | |  | |  | |  | |  | |  | |  | |  | |
| Bcau28 \| *B. caudata* \| Sabah, P. Malaysia | 4.67 | 4.67 | 4.67 | 4.67 | 4.67 | 4.67 | 4.68 | | 0.00 | | 0.00 | | 0.00 | | 0.00 | | 0.00 | | 0.00 | | 0.00 | | 0.00 | | 0.00 | | - | |  | |  | |  | |  | |  | |  | |  | |  | |  | |  | |  | |  | |  | |  | |  | |  | |
| Bcau19 \| *B. caudata* \| Penang, P. Malaysia | 4.67 | 4.67 | 4.67 | 4.67 | 4.67 | 4.67 | 4.68 | | 0.00 | | 0.00 | | 0.00 | | 0.00 | | 0.00 | | 0.00 | | 0.00 | | 0.00 | | 0.00 | | 0.00 | | - | |  | |  | |  | |  | |  | |  | |  | |  | |  | |  | |  | |  | |  | |  | |  | |
| Bcau18 \| *B. caudata* \| University Malaya, P. Malaysia | 4.67 | 4.67 | 4.67 | 4.67 | 4.67 | 4.67 | 4.68 | | 0.00 | | 0.00 | | 0.00 | | 0.00 | | 0.00 | | 0.00 | | 0.00 | | 0.00 | | 0.00 | | 0.00 | | 0.00 | | - | |  | |  | |  | |  | |  | |  | |  | |  | |  | |  | |  | |  | |  | |  | |
| Bcau25 \| *B. caudata* \| Korat, Thailand | 5.48 | 5.48 | 5.48 | 5.48 | 5.48 | 5.48 | 5.50 | 0.83 | | 0.83 | | 0.83 | | 0.83 | | 0.83 | | 0.83 | | 0.83 | | 0.83 | | 0.83 | | 0.83 | | 0.83 | | 0.83 | | - | |  | |  | |  | |  | |  | |  | |  | |  | |  | |  | |  | |  | |  | |  |
| Bcau14 \| *B. caudata* \| Carey Island, P. Malaysia | 4.39 | 4.39 | 4.39 | 4.39 | 4.39 | 4.39 | 4.40 | 0.28 | | 0.28 | | 0.28 | | 0.28 | | 0.28 | | 0.28 | | 0.28 | | 0.28 | | 0.28 | | 0.28 | | 0.28 | | 0.28 | | 1.11 | | - | |  | |  | |  | |  | |  | |  | |  | |  | |  | |  | |  | |  | |  |
| Bcau20 \| *B. caudata* \| Penang, P. Malaysia | 4.39 | 4.39 | 4.39 | 4.39 | 4.39 | 4.39 | 4.40 | 0.28 | | 0.28 | | 0.28 | | 0.28 | | 0.28 | | 0.28 | | 0.28 | | 0.28 | | 0.28 | | 0.28 | | 0.28 | | 0.28 | | 1.11 | | 0.00 | | - | |  | |  | |  | |  | |  | |  | |  | |  | |  | |  | |  | |  |
| Bcau4 \| *B. caudata* \| Clearwater, P. Malaysia | 4.39 | 4.39 | 4.39 | 4.39 | 4.39 | 4.39 | 4.40 | 0.28 | | 0.28 | | 0.28 | | 0.28 | | 0.28 | | 0.28 | | 0.28 | | 0.28 | | 0.28 | | 0.28 | | 0.28 | | 0.28 | | 1.11 | | 0.00 | | 0.00 | | - | |  | |  | |  | |  | |  | |  | |  | |  | |  | |  | |  |
| Bcau9 \| *B. caudata* \| Pahang, Mentakab | 4.39 | 4.39 | 4.39 | 4.39 | 4.39 | 4.39 | 4.40 | 0.28 | | 0.28 | | 0.28 | | 0.28 | | 0.28 | | 0.28 | | 0.28 | | 0.28 | | 0.28 | | 0.28 | | 0.28 | | 0.28 | | 1.11 | | 0.00 | | 0.00 | | 0.00 | | - | |  | |  | |  | |  | |  | |  | |  | |  | |  | |  |
| Bcau8 \| *B. caudata* \| Clearwater, P. Malaysia | 4.39 | 4.39 | 4.39 | 4.39 | 4.39 | 4.39 | 4.40 | 0.28 | | 0.28 | | 0.28 | | 0.28 | | 0.28 | | 0.28 | | 0.28 | | 0.28 | | 0.28 | | 0.28 | | 0.28 | | 0.28 | | 1.11 | | 0.00 | | 0.00 | | 0.00 | | 0.00 | | - | |  | |  | |  | |  | |  | |  | |  | |  | |  |
| Bcau11 \| *B. caudata* \| Carey Island, P. Malaysia | 4.39 | 4.39 | 4.39 | 4.39 | 4.39 | 4.39 | 4.40 | 0.28 | | 0.28 | | 0.28 | | 0.28 | | 0.28 | | 0.28 | | 0.28 | | 0.28 | | 0.28 | | 0.28 | | 0.28 | | 0.28 | | 1.11 | | 0.00 | | 0.00 | | 0.00 | | 0.00 | | 0.00 | | - | |  | |  | |  | |  | |  | |  | |  | |  |
| Bcau10 \| *B. caudata* \| Clearwater, P. Malaysia | 4.39 | 4.39 | 4.39 | 4.39 | 4.39 | 4.39 | 4.40 | 0.28 | | 0.28 | | 0.28 | | 0.28 | | 0.28 | | 0.28 | | 0.28 | | 0.28 | | 0.28 | | 0.28 | | 0.28 | | 0.28 | | 1.11 | | 0.00 | | 0.00 | | 0.00 | | 0.00 | | 0.00 | | 0.00 | | - | |  | |  | |  | |  | |  | |  | |  |
| Bcau2 \| *B. caudata* \| University Malaya, P. Malaysia | 4.39 | 4.39 | 4.39 | 4.39 | 4.39 | 4.39 | 4.40 | 0.28 | | 0.28 | | 0.28 | | 0.28 | | 0.28 | | 0.28 | | 0.28 | | 0.28 | | 0.28 | | 0.28 | | 0.28 | | 0.28 | | 1.11 | | 0.00 | | 0.00 | | 0.00 | | 0.00 | | 0.00 | | 0.00 | | 0.00 | | - | |  | |  | |  | |  | |  | |  |
| Bcau3 \| *B. caudata* \| Clearwater, P. Malaysia | 4.39 | 4.39 | 4.39 | 4.39 | 4.39 | 4.39 | 4.40 | 0.28 | | 0.28 | | 0.28 | | 0.28 | | 0.28 | | 0.28 | | 0.28 | | 0.28 | | 0.28 | | 0.28 | | 0.28 | | 0.28 | | 1.11 | | 0.00 | | 0.00 | | 0.00 | | 0.00 | | 0.00 | | 0.00 | | 0.00 | | 0.00 | | - | |  | |  | |  | |  | |  |
| Bcau7 \| *B. caudata* \| Marang, P. Malaysia | 4.39 | 4.39 | 4.39 | 4.39 | 4.39 | 4.39 | 4.40 | 0.28 | | 0.28 | | 0.28 | | 0.28 | | 0.28 | | 0.28 | | 0.28 | | 0.28 | | 0.28 | | 0.28 | | 0.28 | | 0.28 | | 1.11 | | 0.00 | | 0.00 | | 0.00 | | 0.00 | | 0.00 | | 0.00 | | 0.00 | | 0.00 | | 0.00 | | - | |  | |  | |  | |  |
| Bcau24 \| *B. caudata* \| Tioman Island, P. Malaysia | 4.12 | 4.12 | 4.12 | 4.12 | 4.12 | 4.12 | 4.13 | 0.55 | | 0.55 | | 0.55 | | 0.55 | | 0.55 | | 0.55 | | 0.55 | | 0.55 | | 0.55 | | 0.55 | | 0.55 | | 0.55 | | 1.38 | | 0.28 | | 0.28 | | 0.28 | | 0.28 | | 0.28 | | 0.28 | | 0.28 | | 0.28 | | 0.28 | | 0.28 | | - | |  | |  | |  |
| AY037406 \| *B. caudata* \| Bandar Seri Begawan, Brunei | 4.41 | 4.41 | 4.41 | 4.41 | 4.41 | 4.41 | 4.42 | 0.83 | | 0.83 | | 0.83 | | 0.83 | | 0.83 | | 0.83 | | 0.83 | | 0.83 | | 0.83 | | 0.83 | | 0.83 | | 0.83 | | 1.66 | | 0.55 | | 0.55 | | 0.55 | | 0.55 | | 0.55 | | 0.55 | | 0.55 | | 0.55 | | 0.55 | | 0.55 | | 0.28 | | - | |  | |  |

Table H. Percentage of uncorrected “p” distance matrix of *Bactocera caudata* from various geographical locations in northern and southern hemispheres based on 16SrDNA.

|  | 1 | 2 | 3 | 4 | 5 | 6 | 7 | 8 | 9 | 10 | 11 | 12 | 13 | 14 | 15 | 16 | 17 | 18 | 19 | 20 | 21 | 22 | 23 | 24 | 25 | 26 | 27 | 28 | 29 | 30 | 31 | 32 | 33 |
| --- | --- | --- | --- | --- | --- | --- | --- | --- | --- | --- | --- | --- | --- | --- | --- | --- | --- | --- | --- | --- | --- | --- | --- | --- | --- | --- | --- | --- | --- | --- | --- | --- | --- |
| JN542425 \| *B. caudata* \| Bali, Indonesia | - |  |  |  |  |  |  |  |  |  |  |  |  |  |  |  |  |  |  |  |  |  |  |  |  |  |  |  |  |  |  |  |  |
| JN542424 \| *B. caudata* \| Lombok, Indonesia | 0.00 | - |  |  |  |  |  |  |  |  |  |  |  |  |  |  |  |  |  |  |  |  |  |  |  |  |  |  |  |  |  |  |  |
| Bcau17 \| *B. caudata* \| Lombok, Indonesia | 0.00 | 0.00 | - |  |  |  |  |  |  |  |  |  |  |  |  |  |  |  |  |  |  |  |  |  |  |  |  |  |  |  |  |  |  |
| Bcau31 \| *B. caudata* \| Java, Indonesia | 0.00 | 0.00 | 0.00 | - |  |  |  |  |  |  |  |  |  |  |  |  |  |  |  |  |  |  |  |  |  |  |  |  |  |  |  |  |  |
| Bcau30 \| *B. caudata* \| Java, Indonesia | 0.00 | 0.00 | 0.00 | 0.00 | - |  |  |  |  |  |  |  |  |  |  |  |  |  |  |  |  |  |  |  |  |  |  |  |  |  |  |  |  |
| Bcau13 \| *B. caudata* \| Lombok, Indonesia | 0.00 | 0.00 | 0.00 | 0.00 | 0.00 | - |  |  |  |  |  |  |  |  |  |  |  |  |  |  |  |  |  |  |  |  |  |  |  |  |  |  |  |
| Bcau29 \| *B. caudata \|* Java, Indonesia | 0.00 | 0.00 | 0.00 | 0.00 | 0.00 | 0.00 | - |  |  |  |  |  |  |  |  |  |  |  |  |  |  |  |  |  |  |  |  |  |  |  |  |  |  |
| JN542422 \| *B. caudata* \| Carey Island, P. Malaysia | 2.99 | 2.99 | 2.99 | 2.99 | 2.99 | 2.99 | 2.99 | - |  |  |  |  |  |  |  |  |  |  |  |  |  |  |  |  |  |  |  |  |  |  |  |  |  |
| AY037363 \| *B. caudata* \| Bandar Seri Begawan, Brunei | 2.76 | 2.76 | 2.76 | 2.76 | 2.76 | 2.76 | 2.76 | 0.23 | - |  |  |  |  |  |  |  |  |  |  |  |  |  |  |  |  |  |  |  |  |  |  |  |  |
| JN542423 \| *B. caudata* \| University Malaya, P. Malaysia | 2.76 | 2.76 | 2.76 | 2.76 | 2.76 | 2.76 | 2.76 | 0.23 | 0.00 | - |  |  |  |  |  |  |  |  |  |  |  |  |  |  |  |  |  |  |  |  |  |  |  |
| Bcau11 \| *B. caudata* \| Carey Island, P. Malaysia | 2.76 | 2.76 | 2.76 | 2.76 | 2.76 | 2.76 | 2.76 | 0.23 | 0.00 | 0.00 | - |  |  |  |  |  |  |  |  |  |  |  |  |  |  |  |  |  |  |  |  |  |  |
| Bcau9 \| *B. caudata* \| Mentakab, P. Malaysia | 2.76 | 2.76 | 2.76 | 2.76 | 2.76 | 2.76 | 2.76 | 0.23 | 0.00 | 0.00 | 0.00 | - |  |  |  |  |  |  |  |  |  |  |  |  |  |  |  |  |  |  |  |  |  |
| Bcau7 \| *B. caudata* \| Marang, P. Malaysia | 2.76 | 2.76 | 2.76 | 2.76 | 2.76 | 2.76 | 2.76 | 0.23 | 0.00 | 0.00 | 0.00 | 0.00 | - |  |  |  |  |  |  |  |  |  |  |  |  |  |  |  |  |  |  |  |  |
| Bcau19 \| *B. caudata* \| Penang, P. Malaysia | 2.76 | 2.76 | 2.76 | 2.76 | 2.76 | 2.76 | 2.76 | 0.23 | 0.00 | 0.00 | 0.00 | 0.00 | 0.00 | - |  |  |  |  |  |  |  |  |  |  |  |  |  |  |  |  |  |  |  |
| Bcau8 \| *B. caudata* \| Clearwater, P. Malaysia | 2.76 | 2.76 | 2.76 | 2.76 | 2.76 | 2.76 | 2.76 | 0.23 | 0.00 | 0.00 | 0.00 | 0.00 | 0.00 | 0.00 | - |  |  |  |  |  |  |  |  |  |  |  |  |  |  |  |  |  |  |
| Bcau5 \| *B. caudata* \| Clearwater, P. Malaysia | 2.76 | 2.76 | 2.76 | 2.76 | 2.76 | 2.76 | 2.76 | 0.23 | 0.00 | 0.00 | 0.00 | 0.00 | 0.00 | 0.00 | 0.00 | - |  |  |  |  |  |  |  |  |  |  |  |  |  |  |  |  |  |
| Bcau1 \| *B. caudata* \| University Malaya, P. Malaysia | 2.76 | 2.76 | 2.76 | 2.76 | 2.76 | 2.76 | 2.76 | 0.23 | 0.00 | 0.00 | 0.00 | 0.00 | 0.00 | 0.00 | 0.00 | 0.00 | - |  |  |  |  |  |  |  |  |  |  |  |  |  |  |  |  |
| Bcau16 \| *B. caudata* \| Gombak, P. Malaysia | 2.76 | 2.76 | 2.76 | 2.76 | 2.76 | 2.76 | 2.76 | 0.23 | 0.00 | 0.00 | 0.00 | 0.00 | 0.00 | 0.00 | 0.00 | 0.00 | 0.00 | - |  |  |  |  |  |  |  |  |  |  |  |  |  |  |  |
| Bcau27 \| *B. caudata* \| Selakan, E. Malaysia | 2.76 | 2.76 | 2.76 | 2.76 | 2.76 | 2.76 | 2.76 | 0.23 | 0.00 | 0.00 | 0.00 | 0.00 | 0.00 | 0.00 | 0.00 | 0.00 | 0.00 | 0.00 | - |  |  |  |  |  |  |  |  |  |  |  |  |  |  |
| Bcau10 \| *B. caudata* \| Clearwater, P. Malaysia | 2.76 | 2.76 | 2.76 | 2.76 | 2.76 | 2.76 | 2.76 | 0.23 | 0.00 | 0.00 | 0.00 | 0.00 | 0.00 | 0.00 | 0.00 | 0.00 | 0.00 | 0.00 | 0.00 | - |  |  |  |  |  |  |  |  |  |  |  |  |  |
| Bcau24 \| *B. caudata* \| Tioman Island, P. Malaysia | 2.76 | 2.76 | 2.76 | 2.76 | 2.76 | 2.76 | 2.76 | 0.23 | 0.00 | 0.00 | 0.00 | 0.00 | 0.00 | 0.00 | 0.00 | 0.00 | 0.00 | 0.00 | 0.00 | 0.00 | - |  |  |  |  |  |  |  |  |  |  |  |  |
| Bcau20 \| *B. caudata* \| Penang, P. Malaysia | 2.76 | 2.76 | 2.76 | 2.76 | 2.76 | 2.76 | 2.76 | 0.23 | 0.00 | 0.00 | 0.00 | 0.00 | 0.00 | 0.00 | 0.00 | 0.00 | 0.00 | 0.00 | 0.00 | 0.00 | 0.00 | - |  |  |  |  |  |  |  |  |  |  |  |
| Bcau18 \| *B. caudata* \| University Malaya, P. Malaysia | 2.76 | 2.76 | 2.76 | 2.76 | 2.76 | 2.76 | 2.76 | 0.23 | 0.00 | 0.00 | 0.00 | 0.00 | 0.00 | 0.00 | 0.00 | 0.00 | 0.00 | 0.00 | 0.00 | 0.00 | 0.00 | 0.00 | - |  |  |  |  |  |  |  |  |  |  |
| Bcau25 \| *B. caudata* \| Korat, P. Malaysia | 2.76 | 2.76 | 2.76 | 2.76 | 2.76 | 2.76 | 2.76 | 0.23 | 0.00 | 0.00 | 0.00 | 0.00 | 0.00 | 0.00 | 0.00 | 0.00 | 0.00 | 0.00 | 0.00 | 0.00 | 0.00 | 0.00 | 0.00 | - |  |  |  |  |  |  |  |  |  |
| Bcau4 \| *B. caudata* \| Clearwater, P. Malaysia | 2.76 | 2.76 | 2.76 | 2.76 | 2.76 | 2.76 | 2.76 | 0.23 | 0.00 | 0.00 | 0.00 | 0.00 | 0.00 | 0.00 | 0.00 | 0.00 | 0.00 | 0.00 | 0.00 | 0.00 | 0.00 | 0.00 | 0.00 | 0.00 | - |  |  |  |  |  |  |  |  |
| Bcau21 \| *B. caudata* \| Penang, P. Malaysia | 2.76 | 2.76 | 2.76 | 2.76 | 2.76 | 2.76 | 2.76 | 0.23 | 0.00 | 0.00 | 0.00 | 0.00 | 0.00 | 0.00 | 0.00 | 0.00 | 0.00 | 0.00 | 0.00 | 0.00 | 0.00 | 0.00 | 0.00 | 0.00 | 0.00 | - |  |  |  |  |  |  |  |
| Bcau22 \| *B. caudata* \| Tioman Island, P. Malaysia | 2.76 | 2.76 | 2.76 | 2.76 | 2.76 | 2.76 | 2.76 | 0.23 | 0.00 | 0.00 | 0.00 | 0.00 | 0.00 | 0.00 | 0.00 | 0.00 | 0.00 | 0.00 | 0.00 | 0.00 | 0.00 | 0.00 | 0.00 | 0.00 | 0.00 | 0.00 | - |  |  |  |  |  |  |
| Bcau28 \| *B. caudata* \| Semporna, P. Malaysia | 2.76 | 2.76 | 2.76 | 2.76 | 2.76 | 2.76 | 2.76 | 0.23 | 0.00 | 0.00 | 0.00 | 0.00 | 0.00 | 0.00 | 0.00 | 0.00 | 0.00 | 0.00 | 0.00 | 0.00 | 0.00 | 0.00 | 0.00 | 0.00 | 0.00 | 0.00 | 0.00 | - |  |  |  |  |  |
| Bcau3 \| *B. caudata* \| Clearwater, P. Malaysia | 2.76 | 2.76 | 2.76 | 2.76 | 2.76 | 2.76 | 2.76 | 0.23 | 0.00 | 0.00 | 0.00 | 0.00 | 0.00 | 0.00 | 0.00 | 0.00 | 0.00 | 0.00 | 0.00 | 0.00 | 0.00 | 0.00 | 0.00 | 0.00 | 0.00 | 0.00 | 0.00 | 0.00 | - |  |  |  |  |
| Bcau23 \| *B. caudata* \| University Malaya, P. Malaysia | 2.76 | 2.76 | 2.76 | 2.76 | 2.76 | 2.76 | 2.76 | 0.23 | 0.00 | 0.00 | 0.00 | 0.00 | 0.00 | 0.00 | 0.00 | 0.00 | 0.00 | 0.00 | 0.00 | 0.00 | 0.00 | 0.00 | 0.00 | 0.00 | 0.00 | 0.00 | 0.00 | 0.00 | 0.00 | - |  |  |  |
| Bcau26 \| *B. caudata* \| Gombak, P. Malaysia | 2.76 | 2.76 | 2.76 | 2.76 | 2.76 | 2.76 | 2.76 | 0.23 | 0.00 | 0.00 | 0.00 | 0.00 | 0.00 | 0.00 | 0.00 | 0.00 | 0.00 | 0.00 | 0.00 | 0.00 | 0.00 | 0.00 | 0.00 | 0.00 | 0.00 | 0.00 | 0.00 | 0.00 | 0.00 | 0.00 | - |  |  |
| Bcau32 \| *B. caudata* \| Jemaluang, P. Malaysia | 2.76 | 2.76 | 2.76 | 2.76 | 2.76 | 2.76 | 2.76 | 0.23 | 0.00 | 0.00 | 0.00 | 0.00 | 0.00 | 0.00 | 0.00 | 0.00 | 0.00 | 0.00 | 0.00 | 0.00 | 0.00 | 0.00 | 0.00 | 0.00 | 0.00 | 0.00 | 0.00 | 0.00 | 0.00 | 0.00 | 0.00 | - |  |

Table I. Percentage of uncorrected “p” distance matrix of *Bactocera caudata* from various geographical locations in northern and southern hemispheres based on 28S rDNA.

|  | 1 | 2 | 3 | 4 | 5 | 6 | 7 | 8 | 9 | 10 | 11 | 12 | 13 | 14 | 15 | 16 | 17 | 18 | 19 | 20 | 21 | 22 | 23 | 24 | 25 | 26 | 27 | 28 | 29 | 30 | 31 | 32 | 33 |
| --- | --- | --- | --- | --- | --- | --- | --- | --- | --- | --- | --- | --- | --- | --- | --- | --- | --- | --- | --- | --- | --- | --- | --- | --- | --- | --- | --- | --- | --- | --- | --- | --- | --- |
| Bcau16 \| *B. caudata* \| Gombak, P. Malaysia | - |  |  |  |  |  |  |  |  |  |  |  |  |  |  |  |  |  |  |  |  |  |  |  |  |  |  |  |  |  |  |  |  |
| Bcau19 \| *B. caudata* \| Penang, P. Malaysia | 0.00 | - |  |  |  |  |  |  |  |  |  |  |  |  |  |  |  |  |  |  |  |  |  |  |  |  |  |  |  |  |  |  |  |
| Bcau14 \| *B. caudata* \| Carey Island P. Malaysia | 0.12 | 0.12 | - |  |  |  |  |  |  |  |  |  |  |  |  |  |  |  |  |  |  |  |  |  |  |  |  |  |  |  |  |  |  |
| Bcau4 \| *B. caudata* \| Clearwater P. Malaysia | 0.12 | 0.12 | 0.00 | - |  |  |  |  |  |  |  |  |  |  |  |  |  |  |  |  |  |  |  |  |  |  |  |  |  |  |  |  |  |
| Bcau23 \| *B. caudata* \| University Malaya P. Malaysia | 0.12 | 0.12 | 0.00 | 0.00 | - |  |  |  |  |  |  |  |  |  |  |  |  |  |  |  |  |  |  |  |  |  |  |  |  |  |  |  |  |
| Bcau24 \| *B. caudata* \| Tioman Island P. Malaysia | 0.12 | 0.12 | 0.00 | 0.00 | 0.00 | - |  |  |  |  |  |  |  |  |  |  |  |  |  |  |  |  |  |  |  |  |  |  |  |  |  |  |  |
| Bcau17 \| *B. caudata* \| Lombok, Indonesia | 0.12 | 0.12 | 0.00 | 0.00 | 0.00 | 0.00 | - |  |  |  |  |  |  |  |  |  |  |  |  |  |  |  |  |  |  |  |  |  |  |  |  |  |  |
| Bcau11 \| *B. caudata* \| Carey Island P. Malaysia | 0.12 | 0.12 | 0.00 | 0.00 | 0.00 | 0.00 | 0.00 | - |  |  |  |  |  |  |  |  |  |  |  |  |  |  |  |  |  |  |  |  |  |  |  |  |  |
| Bcau28 \| *B. caudata* \| Semporna, E. Malaysia | 0.12 | 0.12 | 0.00 | 0.00 | 0.00 | 0.00 | 0.00 | 0.00 | - |  |  |  |  |  |  |  |  |  |  |  |  |  |  |  |  |  |  |  |  |  |  |  |  |
| Bcau27 \| *B. caudata* \| Sabah, East Malaysia | 0.12 | 0.12 | 0.00 | 0.00 | 0.00 | 0.00 | 0.00 | 0.00 | 0.00 | - |  |  |  |  |  |  |  |  |  |  |  |  |  |  |  |  |  |  |  |  |  |  |  |
| Bcau22 \| *B. caudata* \| Tioman Island, East Malaysia | 0.12 | 0.12 | 0.00 | 0.00 | 0.00 | 0.00 | 0.00 | 0.00 | 0.00 | 0.00 | - |  |  |  |  |  |  |  |  |  |  |  |  |  |  |  |  |  |  |  |  |  |  |
| Bcau21 \| *B. caudata* \| Penang P. Malaysia | 0.12 | 0.12 | 0.00 | 0.00 | 0.00 | 0.00 | 0.00 | 0.00 | 0.00 | 0.00 | 0.00 | - |  |  |  |  |  |  |  |  |  |  |  |  |  |  |  |  |  |  |  |  |  |
| Bcau9 \| *B. caudata* \| Mentakab, P. Malaysia | 0.12 | 0.12 | 0.00 | 0.00 | 0.00 | 0.00 | 0.00 | 0.00 | 0.00 | 0.00 | 0.00 | 0.00 | - |  |  |  |  |  |  |  |  |  |  |  |  |  |  |  |  |  |  |  |  |
| Bcau15 \| *B. caudata* \|Bali, Indonesia | 0.12 | 0.12 | 0.00 | 0.00 | 0.00 | 0.00 | 0.00 | 0.00 | 0.00 | 0.00 | 0.00 | 0.00 | 0.00 | - |  |  |  |  |  |  |  |  |  |  |  |  |  |  |  |  |  |  |  |
| Bcau31 \| *B. caudata* \| Java, Indonesia | 0.12 | 0.12 | 0.00 | 0.00 | 0.00 | 0.00 | 0.00 | 0.00 | 0.00 | 0.00 | 0.00 | 0.00 | 0.00 | 0.00 | - |  |  |  |  |  |  |  |  |  |  |  |  |  |  |  |  |  |  |
| Bcau30 \| *B. caudata* \| Java, Indonesia | 0.12 | 0.12 | 0.00 | 0.00 | 0.00 | 0.00 | 0.00 | 0.00 | 0.00 | 0.00 | 0.00 | 0.00 | 0.00 | 0.00 | 0.00 | - |  |  |  |  |  |  |  |  |  |  |  |  |  |  |  |  |  |
| Bcau6 \| *B. caudata* \| Lombok, Indonesia | 0.12 | 0.12 | 0.00 | 0.00 | 0.00 | 0.00 | 0.00 | 0.00 | 0.00 | 0.00 | 0.00 | 0.00 | 0.00 | 0.00 | 0.00 | 0.00 | - |  |  |  |  |  |  |  |  |  |  |  |  |  |  |  |  |
| Bcau32 \| *B. caudata* \| Jemaluang P. Malaysia | 0.12 | 0.12 | 0.00 | 0.00 | 0.00 | 0.00 | 0.00 | 0.00 | 0.00 | 0.00 | 0.00 | 0.00 | 0.00 | 0.00 | 0.00 | 0.00 | 0.00 | - |  |  |  |  |  |  |  |  |  |  |  |  |  |  |  |
| Bcau20 \| *B. caudata* \| Penang P. Malaysia | 0.12 | 0.12 | 0.00 | 0.00 | 0.00 | 0.00 | 0.00 | 0.00 | 0.00 | 0.00 | 0.00 | 0.00 | 0.00 | 0.00 | 0.00 | 0.00 | 0.00 | 0.00 | - |  |  |  |  |  |  |  |  |  |  |  |  |  |  |
| Bcau5 \| *B. caudata* \| Clearwater P. Malaysia | 0.12 | 0.12 | 0.00 | 0.00 | 0.00 | 0.00 | 0.00 | 0.00 | 0.00 | 0.00 | 0.00 | 0.00 | 0.00 | 0.00 | 0.00 | 0.00 | 0.00 | 0.00 | 0.00 | - |  |  |  |  |  |  |  |  |  |  |  |  |  |
| Bcau8 \| *B. caudata* \| Clearwater P. Malaysia | 0.12 | 0.12 | 0.00 | 0.00 | 0.00 | 0.00 | 0.00 | 0.00 | 0.00 | 0.00 | 0.00 | 0.00 | 0.00 | 0.00 | 0.00 | 0.00 | 0.00 | 0.00 | 0.00 | 0.00 | - |  |  |  |  |  |  |  |  |  |  |  |  |
| Bcau1 \| *B. caudata* \| University Malaya P. Malaysia | 0.12 | 0.12 | 0.00 | 0.00 | 0.00 | 0.00 | 0.00 | 0.00 | 0.00 | 0.00 | 0.00 | 0.00 | 0.00 | 0.00 | 0.00 | 0.00 | 0.00 | 0.00 | 0.00 | 0.00 | 0.00 | - |  |  |  |  |  |  |  |  |  |  |  |
| Bcau10 \| *B. caudata* \| Clearwater P. Malaysia | 0.12 | 0.12 | 0.00 | 0.00 | 0.00 | 0.00 | 0.00 | 0.00 | 0.00 | 0.00 | 0.00 | 0.00 | 0.00 | 0.00 | 0.00 | 0.00 | 0.00 | 0.00 | 0.00 | 0.00 | 0.00 | 0.00 | - |  |  |  |  |  |  |  |  |  |  |
| Bcau13 \| *B. caudata* \| Lombok, Indonesia | 0.12 | 0.12 | 0.00 | 0.00 | 0.00 | 0.00 | 0.00 | 0.00 | 0.00 | 0.00 | 0.00 | 0.00 | 0.00 | 0.00 | 0.00 | 0.00 | 0.00 | 0.00 | 0.00 | 0.00 | 0.00 | 0.00 | 0.00 | - |  |  |  |  |  |  |  |  |  |
| Bcau7 \| *B. caudata* \| Marang P. Malaysia | 0.12 | 0.12 | 0.00 | 0.00 | 0.00 | 0.00 | 0.00 | 0.00 | 0.00 | 0.00 | 0.00 | 0.00 | 0.00 | 0.00 | 0.00 | 0.00 | 0.00 | 0.00 | 0.00 | 0.00 | 0.00 | 0.00 | 0.00 | 0.00 | - |  |  |  |  |  |  |  |  |
| Bcau2 \| *B. caudata* \| University Malaya P. Malaysia | 0.12 | 0.12 | 0.00 | 0.00 | 0.00 | 0.00 | 0.00 | 0.00 | 0.00 | 0.00 | 0.00 | 0.00 | 0.00 | 0.00 | 0.00 | 0.00 | 0.00 | 0.00 | 0.00 | 0.00 | 0.00 | 0.00 | 0.00 | 0.00 | 0.00 | - |  |  |  |  |  |  |  |
| Bcau3 \| *B. caudata* \| Clearwater P. Malaysia | 0.12 | 0.12 | 0.00 | 0.00 | 0.00 | 0.00 | 0.00 | 0.00 | 0.00 | 0.00 | 0.00 | 0.00 | 0.00 | 0.00 | 0.00 | 0.00 | 0.00 | 0.00 | 0.00 | 0.00 | 0.00 | 0.00 | 0.00 | 0.00 | 0.00 | 0.00 | - |  |  |  |  |  |  |
| Bcau25 \| *B. caudata* \| Korat, Thailand | 0.12 | 0.12 | 0.00 | 0.00 | 0.00 | 0.00 | 0.00 | 0.00 | 0.00 | 0.00 | 0.00 | 0.00 | 0.00 | 0.00 | 0.00 | 0.00 | 0.00 | 0.00 | 0.00 | 0.00 | 0.00 | 0.00 | 0.00 | 0.00 | 0.00 | 0.00 | 0.00 | - |  |  |  |  |  |
| Bcau29 \| *B. caudata \|* Java, Indonesia | 0.12 | 0.12 | 0.00 | 0.00 | 0.00 | 0.00 | 0.00 | 0.00 | 0.00 | 0.00 | 0.00 | 0.00 | 0.00 | 0.00 | 0.00 | 0.00 | 0.00 | 0.00 | 0.00 | 0.00 | 0.00 | 0.00 | 0.00 | 0.00 | 0.00 | 0.00 | 0.00 | 0.00 | - |  |  |  |  |
| Bcau12 \| *B. caudata* \| Lombok, Indonesia | 0.12 | 0.12 | 0.00 | 0.00 | 0.00 | 0.00 | 0.00 | 0.00 | 0.00 | 0.00 | 0.00 | 0.00 | 0.00 | 0.00 | 0.00 | 0.00 | 0.00 | 0.00 | 0.00 | 0.00 | 0.00 | 0.00 | 0.00 | 0.00 | 0.00 | 0.00 | 0.00 | 0.00 | 0.00 | - |  |  |  |
| Btau28 \| *B. tau* \| University Malaya P. Malaysia | 0.25 | 0.25 | 0.25 | 0.25 | 0.25 | 0.25 | 0.25 | 0.25 | 0.25 | 0.25 | 0.25 | 0.25 | 0.25 | 0.25 | 0.25 | 0.25 | 0.25 | 0.25 | 0.25 | 0.25 | 0.25 | 0.25 | 0.25 | 0.25 | 0.25 | 0.25 | 0.25 | 0.25 | 0.25 | 0.25 | - |  |  |
| Bcau26 \| *B. caudata* \| Gombak P. Malaysia | 0.00 | 0.00 | 0.00 | 0.00 | 0.00 | 0.00 | 0.00 | 0.00 | 0.00 | 0.00 | 0.00 | 0.00 | 0.00 | 0.00 | 0.00 | 0.00 | 0.00 | 0.00 | 0.00 | 0.00 | 0.00 | 0.00 | 0.00 | 0.00 | 0.00 | 0.00 | 0.00 | 0.00 | 0.00 | 0.00 | 0.25 | - |  |
| Bcau18 \| *B. caudata* \| University Malaya P. Malaysia | 0.00 | 0.00 | 0.00 | 0.00 | 0.00 | 0.00 | 0.00 | 0.00 | 0.00 | 0.00 | 0.00 | 0.00 | 0.00 | 0.00 | 0.00 | 0.00 | 0.00 | 0.00 | 0.00 | 0.00 | 0.00 | 0.00 | 0.00 | 0.00 | 0.00 | 0.00 | 0.00 | 0.00 | 0.00 | 0.00 | 0.25 | 0.00 | - |

Table J. Percentage of uncorrected “p” distance matrix of *Bactocera caudata* from various geographical locations in northern and southern hemispheres based on ITS-2.

|  | 1 | 2 | 3 | 4 | 5 | 6 | 7 | 8 | 9 | 10 | 11 | 12 | 13 | 14 | 15 | 16 | 17 | 18 | 19 | 20 | 21 | 22 | 23 | 24 | 25 | 26 | 27 | 28 | 29 | 30 | 31 | 32 |
| --- | --- | --- | --- | --- | --- | --- | --- | --- | --- | --- | --- | --- | --- | --- | --- | --- | --- | --- | --- | --- | --- | --- | --- | --- | --- | --- | --- | --- | --- | --- | --- | --- |
| Bcau26 \| *B. “caudata”* \| Gombak, P.Malaysia | - |  |  |  |  |  |  |  |  |  |  |  |  |  |  |  |  |  |  |  |  |  |  |  |  |  |  |  |  |  |  |  |
| Bcau32 \| *B. “caudata”* \| Jemaluang, P. Malaysia | 0.00 | - |  |  |  |  |  |  |  |  |  |  |  |  |  |  |  |  |  |  |  |  |  |  |  |  |  |  |  |  |  |  |
| Bcau11 \| *B. “caudata”* \| Carey Island, P.Malaysia | 0.23 | 0.23 | - |  |  |  |  |  |  |  |  |  |  |  |  |  |  |  |  |  |  |  |  |  |  |  |  |  |  |  |  |  |
| Bcau16 \| *B. “caudata”* \| Gombak, P.Malaysia | 0.23 | 0.23 | 0.00 | - |  |  |  |  |  |  |  |  |  |  |  |  |  |  |  |  |  |  |  |  |  |  |  |  |  |  |  |  |
| Bcau22 \| *B. “caudata”* \| Tioman Island, P.Malaysia | 0.23 | 0.23 | 0.00 | 0.00 | - |  |  |  |  |  |  |  |  |  |  |  |  |  |  |  |  |  |  |  |  |  |  |  |  |  |  |  |
| Bcau3 \| *B. “caudata”* \| Clearwater, P.Malaysia | 0.23 | 0.23 | 0.00 | 0.00 | 0.00 | - |  |  |  |  |  |  |  |  |  |  |  |  |  |  |  |  |  |  |  |  |  |  |  |  |  |  |
| Bcau20 \| *B. “caudata”* \| Penang, P.Malaysia | 0.23 | 0.23 | 0.00 | 0.00 | 0.00 | 0.00 | - |  |  |  |  |  |  |  |  |  |  |  |  |  |  |  |  |  |  |  |  |  |  |  |  |  |
| Bcau14 \| *B. “caudata”* \| Carey Island, P.Malaysia | 0.23 | 0.23 | 0.00 | 0.00 | 0.00 | 0.00 | 0.00 | - |  |  |  |  |  |  |  |  |  |  |  |  |  |  |  |  |  |  |  |  |  |  |  |  |
| Bcau24 \| *B. “caudata”* \| Tioman Island, P.Malaysia | 0.23 | 0.23 | 0.00 | 0.00 | 0.00 | 0.00 | 0.00 | 0.00 | - |  |  |  |  |  |  |  |  |  |  |  |  |  |  |  |  |  |  |  |  |  |  |  |
| Bcau21 \| *B. “caudata”* \| Penang, P.Malaysia | 0.23 | 0.23 | 0.00 | 0.00 | 0.00 | 0.00 | 0.00 | 0.00 | 0.00 | - |  |  |  |  |  |  |  |  |  |  |  |  |  |  |  |  |  |  |  |  |  |  |
| Bcau25 \| *B. “caudata”* \| Korat, Thailand | 0.23 | 0.23 | 0.00 | 0.00 | 0.00 | 0.00 | 0.00 | 0.00 | 0.00 | 0.00 | - |  |  |  |  |  |  |  |  |  |  |  |  |  |  |  |  |  |  |  |  |  |
| Bcau10 \| *B. “caudata”* \| Clearwater, P.Malaysia | 0.00 | 0.00 | 0.23 | 0.23 | 0.23 | 0.23 | 0.23 | 0.23 | 0.23 | 0.23 | 0.00 | - |  |  |  |  |  |  |  |  |  |  |  |  |  |  |  |  |  |  |  |  |
| Bcau2 \| *B. “caudata”* \| University Malaya, P.Malaysia | 0.00 | 0.00 | 0.23 | 0.23 | 0.23 | 0.23 | 0.23 | 0.23 | 0.23 | 0.23 | 0.00 | 0.00 | - |  |  |  |  |  |  |  |  |  |  |  |  |  |  |  |  |  |  |  |
| Bcau8 \| *B. “caudata”* \| Clearwater, P.Malaysia | 0.00 | 0.00 | 0.23 | 0.23 | 0.23 | 0.23 | 0.23 | 0.23 | 0.23 | 0.23 | 0.00 | 0.00 | 0.00 | - |  |  |  |  |  |  |  |  |  |  |  |  |  |  |  |  |  |  |
| Bcau17 \| *B. caudata* \|Lombok, Indonesia | 0.00 | 0.00 | 0.23 | 0.23 | 0.23 | 0.23 | 0.23 | 0.23 | 0.23 | 0.23 | 0.00 | 0.00 | 0.00 | 0.00 | - |  |  |  |  |  |  |  |  |  |  |  |  |  |  |  |  |  |
| Bcau18 \| *B. “caudata”* \| University Malaya Indonesia | 0.00 | 0.00 | 0.23 | 0.23 | 0.23 | 0.23 | 0.23 | 0.23 | 0.23 | 0.23 | 0.00 | 0.00 | 0.00 | 0.00 | 0.00 | - |  |  |  |  |  |  |  |  |  |  |  |  |  |  |  |  |
| Bcau27 \| *B. “caudata”* \| Selakan, East Malaysia | 0.00 | 0.00 | 0.23 | 0.23 | 0.23 | 0.23 | 0.23 | 0.23 | 0.23 | 0.23 | 0.00 | 0.00 | 0.00 | 0.00 | 0.00 | 0.00 | - |  |  |  |  |  |  |  |  |  |  |  |  |  |  |  |
| Bcau12 \| *B. caudata* \| Lombok, Indonesia | 0.00 | 0.00 | 0.23 | 0.23 | 0.23 | 0.23 | 0.23 | 0.23 | 0.23 | 0.23 | 0.00 | 0.00 | 0.00 | 0.00 | 0.00 | 0.00 | 0.00 | - |  |  |  |  |  |  |  |  |  |  |  |  |  |  |
| Bcau15 \| *B. caudata* \| Bali, Indonesia | 0.00 | 0.00 | 0.23 | 0.23 | 0.23 | 0.23 | 0.23 | 0.23 | 0.23 | 0.23 | 0.00 | 0.00 | 0.00 | 0.00 | 0.00 | 0.00 | 0.00 | 0.00 | - |  |  |  |  |  |  |  |  |  |  |  |  |  |
| Bcau30 \| *B. caudata* \| Java, Indonesia | 0.00 | 0.00 | 0.23 | 0.23 | 0.23 | 0.23 | 0.23 | 0.23 | 0.23 | 0.23 | 0.00 | 0.00 | 0.00 | 0.00 | 0.00 | 0.00 | 0.00 | 0.00 | 0.00 | - |  |  |  |  |  |  |  |  |  |  |  |  |
| Bcau31 \| *B. caudata* \| Java, Indonesia | 0.00 | 0.00 | 0.23 | 0.23 | 0.23 | 0.23 | 0.23 | 0.23 | 0.23 | 0.23 | 0.00 | 0.00 | 0.00 | 0.00 | 0.00 | 0.00 | 0.00 | 0.00 | 0.00 | 0.00 | - |  |  |  |  |  |  |  |  |  |  |  |
| Bcau13 \| *B. caudata* \| Lombok, Indonesia | 0.00 | 0.00 | 0.23 | 0.23 | 0.23 | 0.23 | 0.23 | 0.23 | 0.23 | 0.23 | 0.00 | 0.00 | 0.00 | 0.00 | 0.00 | 0.00 | 0.00 | 0.00 | 0.00 | 0.00 | 0.00 | - |  |  |  |  |  |  |  |  |  |  |
| Bcau29 \| *B. caudata \|* Java, Indonesia | 0.00 | 0.00 | 0.23 | 0.23 | 0.23 | 0.23 | 0.23 | 0.23 | 0.23 | 0.23 | 0.00 | 0.00 | 0.00 | 0.00 | 0.00 | 0.00 | 0.00 | 0.00 | 0.00 | 0.00 | 0.00 | 0.00 | - |  |  |  |  |  |  |  |  |  |
| Bcau1 \| *B. “caudata”* \| University Malaya, P. Malaysia | 0.00 | 0.00 | 0.23 | 0.23 | 0.23 | 0.23 | 0.23 | 0.23 | 0.23 | 0.23 | 0.00 | 0.00 | 0.00 | 0.00 | 0.00 | 0.00 | 0.00 | 0.00 | 0.00 | 0.00 | 0.00 | 0.00 | 0.00 | - |  |  |  |  |  |  |  |  |
| Bcau5 \| *B. “caudata”* \| Clearwater, P. Malaysia | 0.00 | 0.00 | 0.23 | 0.23 | 0.23 | 0.23 | 0.23 | 0.23 | 0.23 | 0.23 | 0.00 | 0.00 | 0.00 | 0.00 | 0.00 | 0.00 | 0.00 | 0.00 | 0.00 | 0.00 | 0.00 | 0.00 | 0.00 | 0.00 | - |  |  |  |  |  |  |  |
| Bcau23 \| *B. “caudata”* \| University Malaya, P. Malaysia | 0.00 | 0.00 | 0.23 | 0.23 | 0.23 | 0.23 | 0.23 | 0.23 | 0.23 | 0.23 | 0.00 | 0.00 | 0.00 | 0.00 | 0.00 | 0.00 | 0.00 | 0.00 | 0.00 | 0.00 | 0.00 | 0.00 | 0.00 | 0.00 | 0.00 | - |  |  |  |  |  |  |
| Bcau9 \| *B. “caudata”* \| Mentakab, P. Malaysia | 0.00 | 0.00 | 0.23 | 0.23 | 0.23 | 0.23 | 0.23 | 0.23 | 0.23 | 0.23 | 0.00 | 0.00 | 0.00 | 0.00 | 0.00 | 0.00 | 0.00 | 0.00 | 0.00 | 0.00 | 0.00 | 0.00 | 0.00 | 0.00 | 0.00 | 0.00 | - |  |  |  |  |  |
| Bcau19 \| *B. “caudata”* \| Penang, P. Malaysia | 0.00 | 0.00 | 0.23 | 0.23 | 0.23 | 0.23 | 0.23 | 0.23 | 0.23 | 0.23 | 0.00 | 0.00 | 0.00 | 0.00 | 0.00 | 0.00 | 0.00 | 0.00 | 0.00 | 0.00 | 0.00 | 0.00 | 0.00 | 0.00 | 0.00 | 0.00 | 0.00 | - |  |  |  |  |
| Bcau4 \| *B. “caudata”* \| Clearwater, P. Malaysia | 0.00 | 0.00 | 0.23 | 0.23 | 0.23 | 0.23 | 0.23 | 0.23 | 0.23 | 0.23 | 0.00 | 0.00 | 0.00 | 0.00 | 0.00 | 0.00 | 0.00 | 0.00 | 0.00 | 0.00 | 0.00 | 0.00 | 0.00 | 0.00 | 0.00 | 0.00 | 0.00 | 0.00 | - |  |  |  |
| Bcau28 \| *B. “caudata”* \| Semporna, E. Malaysia | 0.00 | 0.00 | 0.23 | 0.23 | 0.23 | 0.23 | 0.23 | 0.23 | 0.23 | 0.23 | 0.00 | 0.00 | 0.00 | 0.00 | 0.00 | 0.00 | 0.00 | 0.00 | 0.00 | 0.00 | 0.00 | 0.00 | 0.00 | 0.00 | 0.00 | 0.00 | 0.00 | 0.00 | 0.00 | - |  |  |
| Bcau7 \| *B. “caudata”* \| Marang, P. Malaysia | 0.00 | 0.00 | 0.23 | 0.23 | 0.23 | 0.23 | 0.23 | 0.23 | 0.23 | 0.23 | 0.00 | 0.00 | 0.00 | 0.00 | 0.00 | 0.00 | 0.00 | 0.00 | 0.00 | 0.00 | 0.00 | 0.00 | 0.00 | 0.00 | 0.00 | 0.00 | 0.00 | 0.00 | 0.00 | 0.00 | - |  |
